# Exploring Co-occurring POLE Exonuclease and Non-exonuclease Domain Mutations and Their Impact on Tumor Mutagenicity

**DOI:** 10.1158/2767-9764.CRC-23-0312

**Published:** 2024-01-26

**Authors:** Shreya M. Shah, Elena V. Demidova, Salena Ringenbach, Bulat Faezov, Mark Andrake, Arjun Gandhi, Pilar Mur, Julen Viana-Errasti, Joanne Xiu, Jeffrey Swensen, Laura Valle, Roland L. Dunbrack, Michael J. Hall, Sanjeevani Arora

**Affiliations:** 1 Cancer Prevention and Control Program, Fox Chase Cancer Center, Philadelphia, Pennsylvania.; 2 Science Scholars Program, Temple University, Philadelphia, Pennsylvania.; 3 Institute of Fundamental Medicine and Biology, Kazan Federal University, Kazan, Russian Federation.; 4 Lewis Katz School of Medicine, Temple University, Bethlehem, Pennsylvania.; 5 Program in Cancer Signaling and Microenvironment, Fox Chase Cancer Center, Philadelphia, Pennsylvania.; 6 University College Dublin School of Medicine and Medical Science, Dublin, Ireland.; 7 Hereditary Cancer Program, Catalan Institute of Oncology, IDIBELL, Hospitalet de Llobregat, Barcelona, Spain.; 8 Caris Life Sciences, Phoenix, Arizona.; 9 Department of Clinical Genetics, Fox Chase Cancer Center, Philadelphia, Pennsylvania.; 10 Department of Radiation Oncology, Fox Chase Cancer Center, Philadelphia, Pennsylvania.

## Abstract

*POLE* driver mutations in the exonuclease domain (ExoD driver) are prevalent in several cancers, including colorectal cancer and endometrial cancer, leading to dramatically ultra-high tumor mutation burden (TMB). To understand whether *POLE* mutations that are not classified as drivers (*POLE* Variant) contribute to mutagenesis, we assessed TMB in 447 *POLE*-mutated colorectal cancers, endometrial cancers, and ovarian cancers classified as TMB-high ≥10 mutations/Mb (mut/Mb) or TMB-low <10 mut/Mb. TMB was significantly highest in tumors with “*POLE* ExoD driver plus *POLE* Variant” (colorectal cancer and endometrial cancer, *P* < 0.001; ovarian cancer, *P* < 0.05). TMB increased with additional *POLE* variants (*P* < 0.001), but plateaued at 2, suggesting an association between the presence of these variants and TMB. Integrated analysis of AlphaFold2 POLE models and quantitative stability estimates predicted the impact of multiple POLE variants on POLE functionality. The prevalence of immunogenic neoepitopes was notably higher in the “*POLE* ExoD driver plus *POLE* Variant” tumors. Overall, this study reveals a novel correlation between *POLE* variants in *POLE* ExoD-driven tumors, and ultra-high TMB. Currently, only select pathogenic ExoD mutations with a reliable association with ultra-high TMB inform clinical practice. Thus, these findings are hypothesis-generating, require functional validation, and could potentially inform tumor classification, treatment responses, and clinical outcomes.

**Significance:**

Somatic POLE ExoD driver mutations cause proofreading deficiency that induces high TMB. This study suggests a novel modifier role for POLE variants in POLE ExoD-driven tumors, associated with ultra-high TMB. These data, in addition to future functional studies, may inform tumor classification, therapeutic response, and patient outcomes.

## Introduction

DNA polymerase epsilon (POLE) is an essential mediator of accurate DNA replication, based in part on its roles in DNA synthesis and DNA proofreading (1). *POLE* mutations that impair DNA proofreading lead to increased mutagenesis, and in the germline confer an increased risk of colorectal, endometrial, and other cancers (2–9). Somatic *POLE* mutations affecting proofreading are relatively rare, typically observed in approximately 2%–8% of colorectal cancers and approximately 7%–15% of endometrial cancers, and less commonly in other tumors (3). Tumors harboring *POLE* mutations that lead to proofreading defects are typically ultra-hypermutated [>100 mutations/Mb (mut/Mb)] and have a specific context of mutational signatures (COSMIC signatures 10a and 10b; ref. 10). The increased tumor mutation burden (TMB) in such tumors is typically associated with the benefit from immune checkpoint inhibitor (ICI) therapy (11). Furthermore, recent studies have also shown that specific pathogenic *POLE* mutations are associated with clinical benefit from ICI therapy, but also superior progression-free survival and overall survival (11, 12). This, understanding how *POLE* mutations impact TMB has important clinical implications for patient outcomes and treatment decisions (11, 13, 14).

Somatic mutations associated with enhanced TMB, and proofreading deficiency are typically observed as hotspot mutations in the exonuclease domain (ExoD) of POLE, such as P286R, V411L, S297F, A456P, and S459F (10), considered driver mutations. Current evidence suggests that most mutations located outside the ExoD or those leading to a truncated protein, do not have an impact on the proofreading function of the polymerase or on the tumor mutational landscape. However, *POLE* variants of uncertain significance (VUS), typically in the non-ExoD or non-hotspot regions of ExoD, are sometimes concurrent with a *POLE* ExoD driver mutation and/or microsatellite instability (MSI; refs. 9, 15, 16). Here, we performed a retrospective analysis of 447 genomic profiles of tumors with *POLE* mutations to investigate the effect of co-occurring *POLE* non-pathogenic variants on TMB, and other tumor molecular features, and POLE protein stability. The data presented here are hypothesis-generating and suggest that non-pathogenic *POLE* variants may further increase *POLE* ExoD driver–associated mutation rates and tumor neoantigens.

## Materials and Methods

### Characterization of the Discovery Cohort (Caris Life Sciences)

We conducted a retrospective analysis on the genomic profiles of 1,870 patients with colorectal cancer, 4,481 patients with endometrial cancer, and 8,190 patients with ovarian cancer that underwent genomic profiling by Caris Life Sciences (CLS) as part of their routine comprehensive tumor molecular profiling. This study was conducted in accordance with 45 CFR 46.101(b), and utilized retrospective, deidentified patient data, making it Institutional Review Board (IRB) exempt, with no need for patient consent. All data were obtained through a Data Use Agreement between CLS and Dr. Michael Hall at the Fox Chase Cancer Center (IRB 15-8003).

CLS performed next-generation sequencing on genomic DNA from formalin-fixed paraffin-embedded tumor samples using the NextSeq platform (Illumina, Inc.). Here, 592 whole-gene targets were enriched using a custom-designed SureSelect XT assay (Agilent Technologies); a total of 1.4 MB was assessed. All reported variants were detected with >99% confidence based on allele frequency and amplicon coverage. The average sequencing depth of coverage was >500 and the analytic sensitivity was 5%. In the sequencing panel, splice junctions are covered with mutations observed at ±30 nucleotides from the boundaries of *BRCA1/2* genes and ±10 nucleotides of the other genes. Splicing variants were annotated only for mutations detected in ±2 nucleotides from the exon boundaries. CLS employed standard practices for TMB analysis (17). TMB was measured by counting all non-synonymous missense, nonsense, inframe insertion/deletion, and frameshift mutations found per tumor, and the threshold to define TMB-high (TMB-H) was ≥10 mut/Mb based on the KEYNOTE-158 pembrolizumab trial (18). No normal samples were sequenced, variants previously reported as germline alterations in dbSNP151, Genome Aggregation Database (gnomAD) databases, or benign variants identified by CLS geneticists were excluded from the TMB calculations. All tests have met the Clinical Laboratory Improvement Amendments/College of American Pathologists (CAP) and ISO requirements. Comprehensive MSI profiling was executed using next-generation sequencing, following the guidelines set forth by CAP for MSI and mismatch repair (MMR) testing (19).

CLS provided patient clinical and demographic data that were collected from electronic medical records between June 2016 and June 2019. Clinically reported TMB values from CLS, tumor lineage, primary tumor site, patient diagnosis, specimen location, age, sex, microsatellite stable/ microsatellite instable (MSS/MSI) status, *POLE* variants, and variants in the 592-targeted gene somatic panel were obtained from CLS. The pathogenicity of each *POLE* mutation was annotated on the basis of the American College of Medical Genetics and Genomics designation as “pathogenic,” “likely pathogenic,” “VUS,” “presumed benign,” or “benign” (20). To stratify patients into groups, we used a list of known POLE ExoD drivers (*n* = 20): D275G, P286R, S297F/Y, F367C/L/V, V411L, L424F, P436R/S/Y, M444K/L, A456P, S459F/Y, S461L/P, A465V (10). Total *POLE*-mutated colorectal cancer, endometrial cancer, and ovarian cancer patient tumor count was *n* = 447 (colorectal cancer, *n* = 92; endometrial cancer, *n* = 307; ovarian cancer, *n* = 48). TMB threshold of <10 was used to get MSS TMB-low (TMB-L), MSI TMB-L, or TMB≥10 to get MSS/TMB-H, and MSI/TMB-H. The age distribution of the patients with colorectal cancer, endometrial cancer, and ovarian cancer within each of the four groups was used to determine the percentage of frequency of the mutations within each cohort for colorectal cancer, endometrial cancer, ovarian cancer, and for all the cancers combined. We plotted the age distribution in Graphpad Prism V.9 (https://www.graphpad.com/) and smoothened the curve using Fit Spline (5 knots). The mutational landscape and patient demographic/clinical characteristics (PD-L1 by IHC, MSI comprehensive, age, and sex) of the four groups for each cancer type were plotted using the GenVisR package of R (21). For each cancer type, from the 592-targeted gene panel, we identified the top 10% of mutated genes. These genes were identified by setting a threshold of at least more than 50% of the patients in any Group per cancer type must have a mutation in that specific gene.

### The Cancer Genome Atlas Validation Cohort

Sequencing data from 46 POLE proofreading–deficient The Cancer Genome Atlas (TCGA) tumor samples (independently of tumor type) and their MSI status (endometrial cancer, *n* = 30; colorectal cancer, *n* = 6; breast cancer, *n* = 1; other, *n* = 9), were obtained from cBioPortal (22, 23). The webtool Single Mutational Signatures in Cancer (MuSiCa) was used to calculate the TMB in those tumors (24). Similar to the discovery cohort, *POLE* alterations and the TMB data were used to segregate the data into either “*POLE* ExoD driver” (*n* = 17; endometrial cancer, *n* = 12; colorectal cancer, *n* = 3; other, *n* = 2) or “*POLE* ExoD driver plus *POLE* Variant” (*n* = 29; endometrial cancer, *n* = 18; colorectal cancer, *n* = 3; breast cancer, *n* = 1; other, *n* = 7); these cohorts were compared using a Mann–Whitney statistical test. The analysis was performed by including and excluding data for MSI-high (MSI-H) tumors (*n* = 6).

### Structural Mapping and Functional Analysis of *POLE* Variants of Interest

To understand the possible structural impact of POLE variants, we used structural modeling on three-dimensional models of human POLE. Initially, we created models through traditional template-driven homology modeling (25) based on yeast POLE structures [PDB codes 4M8O (26) and 6WJV (27)]. Subsequently, we employed Rosetta FastRelax methods (28–30), side-chain rebuilding with SCWRL4 (31, 32), and analysis with UCSF Chimera (33) software. To enhance the accuracy of the full-length human protein model, we adopted the AlphaFold2 (AF2) program for protein structure prediction. While AF2 does not require close homology templates (34, 35), we set AF2 to allow us to choose specific templates, such as those for yeast POLE, to improve model conformation with respect to either DNA ligands or other cooperating subunits.

In the case of human POLE, we accounted for accessory subunits observed in the yeast holoenzyme cryo-EM structure, comprising yeast DNA POLE subunits A, B, C, and D [PDB code 6WJV (27)]. Adjusting the depth of the multiple sequence alignments helped produce a model aligning well with the yeast homolog with an RMSD of 1.02 Å^2^ for 1,002 alpha-carbon pairs in the NTD half, as well as the linker and C-Lobe found in a similar position seen in the template structure (PDB:6WJV). As the yeast protein is known to undergo a significant conformational change upon binding to DNA in two major alpha helices in the finger region, we also generated an AF2 model of the N-terminal half of human POLE based on the structure of the yeast POLE crystallized in the presence of a primer-template DNA molecule [PDB code 4M8O (26)]. This model was subjected to the same ΔΔG monomer process to score all the variants that are located in the N-Lobe portion of POLE (amino acids 1 to 1,183).

To assess quantitatively the effects of missense mutations in POLE, we employed applications found in the Rosetta Molecular Modeling Suite (28, 36). The ΔΔG (or Gibbs free energy) values indicate changes in stability, with negative values suggesting increased stability and positive values indicating destabilization. The ΔΔG is given in Rosetta energy units, which have been calibrated to be equivalent to kcal/mol units (28). It should be noted that this calculated change in stability does not always correlate with effects on protein function. These cautions notwithstanding, calculations were performed for more than 170 variants in human POLE found in colorectal cancer, endometrial cancer, and ovarian cancer. We set a cutoff of 1.45 kcal/mol for significant stabilization or destabilization, considering the SD across decoy sets. Highly destabilizing or stabilizing mutations were labeled as significant. This approach combines structural modeling and energy calculations to assess the impact of missense mutations in human POLE, especially in the context of cancer-associated variants. Also see ref. 37 for more detailed methods.

### *POLE* Single Base Substitution Mutational Signatures, and 3-Nucleotide Sequence Context Analysis for *POLE* Variants, and Variants in *KRAS*, *PTEN,* and *PIK3CA*

Single base substitution (SBS) 10a and 10b cosmic mutational signatures for POLE ExoD defects were analyzed as described previously (38). Signatures for the colorectal cancer and the endometrial cancer Group 3 cohorts are shown. For assessing the 3-nucleotide sequence context of mutations, all *POLE* ExoD driver–associated COSMIC mutational signatures (SBS 10a, SBS 10b, SBS 14, and SBS 28) were assessed (39–41). Each of these signatures has a primary mutation which has been described as a “hotspot” (39); SBS 10a is C>A in TCT context; SBS 10b is C>T in the TCG context; SBS 14 is C>A in the NCT context (N is any base); and SBS 28 is T>G in the TTT context. In addition to these primary “hotspots,” all mutations that constitute >1% of the genome signature of interest were counted, capturing 88%–89% of each signature in the analysis.

### Neoantigen Prediction

Missense mutations from the top 10% of mutated genes in Groups 2 and 3 endometrial cancers and colorectal cancers in the CLS data were selected for neoantigen prediction. To specifically contrast the immunogenicity between Groups 2 and 3, we focused on mutations that were exclusive to Group 3. This was based on the criteria that mutations either did not appear in Group 2 or, if they did, appeared no more than once (≤1), and occurred at least twice in Group 3 (≥2). The genes, amino acid change, and sequence (obtained from UniProt) were entered into DeepNeo (42) to determine the HLA class, peptide sequence, MHC binding prediction score, and T-cell reactivity prediction score of associated neoantigens. On the basis of these results, DeepNeo categorized the neoantigens as “immunogenic neoantigen” if both MHC binding and T-cell reactivity ≥0.5, “nonimmunogenic neoantigen” if MHC binding ≥0.5 and T-cell reactivity <0.5, “no biological significance” if MHC binding <0.5 and T-cell reactivity ≥0.5, and “no neoantigen and no immunogenicity” if both MHC binding and T-cell reactivity <0.5. Combining results from DeepNeo, a modified neoantigen burden was calculated as the number of immunogenic neoantigens that were predicted for each tumor. The modified neoantigen burden was combined for colorectal cancer and endometrial cancer data and was compared with TMB data per tumor, graphed in GraphPad Prism. Finally, the immunogenicity of each neoantigen based on HLA class and peptide sequence was validated through an analysis in Neodb (43), which is the largest database for experimentally validated neoantigens, as well as a platform for the discovery of novel neoantigens.

### Statistical Analysis

We used descriptive statistics to determine medians and quartiles for age distributions. The median TMB (mTMB) with range was reported for each individual cohort for each cancer type (colorectal cancer, endometrial cancer, and ovarian cancer) for the CLS dataset. We combined data from multiple cancer types, where appropriate, to report mTMB with range in MSI-H and MSS or MSI-low tumors. We used Mann–Whitney tests, where appropriate, to determine the clinical significance of the mTMB difference between the compared cohorts; ***, *P* < 0.001; **, *P* < 0.01; *, *P* < 0.05; NS, nonsignificant. Where mentioned, corrections for multiple testing were performed for more than two comparisons using the Benjamini–Hochberg FDR test. A correlation between TMB versus neoantigen burden data was conducted by a Spearman rank correlation with *P* < 0.05 considered statistically significant for each comparison. These *P* values were not adjusted for multiple comparisons.

### Data Availability Statement

The deidentified genomic sequencing data are owned by CLS and are not publicly available. The datasets analyzed during the current study are available from the authors upon reasonable request and with permission of CLS. Qualified researchers may contact the corresponding author with request. Finally, the wildtype (WT) AF2 structure models generated in this study are available at https://zenodo.org/record/7395412#.Y44AwOzMJqs (DOI 10.5281/zenodo.7395412).

## Results

### *POLE* Variants in Colorectal, Endometrial, and Ovarian Tumors

**FIGURE 1:**
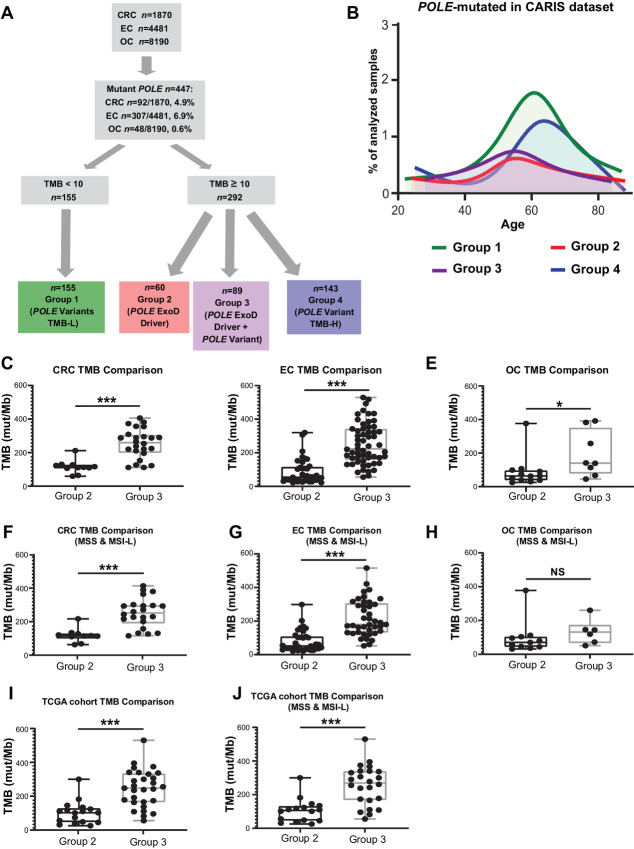
Characterization of *POLE* mutations in the CLS and TCGA dataset. **A,** Flowchart and analysis tree for colorectal cancer (CRC), endometrial cancer (EC), and ovarian cancer (OC) tumors by *POLE* mutations, TMB, and MSI/MSS status. Among 1,870 colorectal cancer, 4,481 endometrial cancers, and 8,910 ovarian cancer tumor genomic profiles, a total of 447 carried *POLE* mutations. Clinically relevant TMB cut-off points were used to define the TMB-H (≥10 mut/Mb) and TMB-L (<10 mut/Mb) cohorts. *POLE* mutation cohorts along with TMB and MSI/MSS status were defined. TMB-L tumors with *POLE* variants but no established *POLE* ExoD driver are referred to as “*POLE* Variants TMB-L” (Group 1, MSS or MSI). TMB-H tumors with known *POLE* ExoD driver only were referred to as “*POLE* ExoD Driver” (Group 2, MSS or MSI). TMB-H tumors with co-occurring *POLE* ExoD driver and *POLE* variant(s) were referred to as “*POLE* ExoD Driver + *POLE* ExoD Variant” (Group 3, MSS or MSI). TMB-H tumors with only *POLE* variant(s) and no *POLE* ExoD driver were referred to as “*POLE* Variant TMB-H” (Group 4, MSS or MSI). **B,** Age distribution of patients in the CLS cohort with *POLE*-mutated tumors (*n* = 447) designated as Group 1 (green), Group 2 (red), Group 3 (purple), and Group 4 (blue). mTMB comparisons between Group 2 and 3 colorectal cancers (**C**), endometrial cancers (**D**), and ovarian cancers (**E**). mTMB comparisons between Group 2 and 3 genomic profiles of colorectal cancer (**F**), endometrial cancer (**G**), and ovarian cancer (**H**). MSI-H tumor profiles were removed from this analysis. TCGA cohort mTMB comparisons between Group 2 and 3 tumors, in **I** MSI-H or MSS tumor profiles were included and in **J** only MSS tumor profiles were included. Because of smaller sample size per tumor type, analyses were pooled. A Mann–Whitney test was performed and ***, *P* < 0.001; *, *P* < 0.05; NS, nonsignificant.

The clinical and demographic characteristics for patients with colorectal cancer, endometrial cancer, and ovarian cancer genomically profiled for *POLE*, TMB, and (where relevant) MSI/MSS status by CLS are in Table 1. *POLE* mutations were observed in 4.9% of colorectal cancers (92/1,870), 6.9% of endometrial cancers (307/4,481), and 0.6% of ovarian cancers (48/8,190; Fig. 1A).

**TABLE 1:** *POLE* variant groups, patient clinical and demographic characteristics for the CARIS cohort

		*POLE* ExoD driver TMB-H		
Clinicopathologic factors	*POLE* variant TMB-L (Group 1)	*POLE* ExoD driver (Group 2)	*POLE* ExoD driver + *POLE* Variant (Group 3)	*POLE* Variant TMB-H (Group 4)
**Colorectal cancer**				
No. of patients	36/92	11/92	24/92	21/92
mTMB	6.0 (3–9)	115 (61–216)	264.5(114–414)	33 (10–461)
MSS	36	11	21	11
MSI	0	0	3	10
<50 yrs	6	5	14	8
≥50 yrs	30	6	10	13
Female	19	1	6	8
Male	17	10	18	13
**Endometrial cancer**				
No. of patients	95/307	37/307	57/307	118/307
mTMB	7 (3–9)	52 (21–314)	219 (53–520)	17.50 (10–273)
MSS	90	35	41	23
MSI	5	2	16	95
<50 yrs	6	6	10	5
≥50 yrs	89	31	47	113
**Ovarian cancer**				
No. of patients	24/48	12/48	8/48	4/48
mTMB	5.0 (4–9)	69 (31–379)	145 (51–394)	14.50 (10–21)
MSS	24	12	6	2
MSI	0	0	2	2
<50 yrs	4	4	5	3
≥50 yrs	20	8	3	1

Abbreviations: MMR, mismatch repair; MSI, microsatellite instability; MSS, microsatellite stability; mTMB, median tumor mutational burden; yrs, years.

Within this dataset of tumors harboring *POLE* mutations (*n* = 447/14,541), low TMB (TMB-L, <10 mut/Mb) was observed in 39.1% of colorectal cancers (36/92), 30.9% of endometrial cancers (95/307), and 50.0% of ovarian cancers (24/48; Fig. 1A; Table 1). TMB-L tumors had *POLE* variants but no established *POLE* ExoD driver mutations (as defined in ref. 10 and Materials and Methods). This group is subsequently referred to as “*POLE* variants TMB-L” (Group 1; Fig. 1A; Table 1; Supplementary Table S1). In contrast, high TMB (TMB-H, ≥10 mut/Mb) was observed in 60.9% of colorectal cancers (56/92), 69.1% of endometrial cancers (212/307), and 50.0% of ovarian cancers (24/48; Fig. 1A; Table 1). TMB-H tumors could be segregated into three groups, subsequently referred to as “*POLE* ExoD driver” (Group 2), “*POLE* ExoD driver plus *POLE* Variant” (Group 3), and “*POLE* Variant TMB-H” group (Group 4, lacking an established ExoD driver; Table 1; Fig. 1A; Supplementary Table S1). The Group 2 and 3 tumors typically had a single established *POLE* ExoD driver; however, five tumors had more than one (Supplementary Table S1). Typically, Groups 2 and 3 occurred in younger patients, while Groups 1 and 4 tumors occurred more frequently in older patients (median ages at diagnosis: 55.5, 55, 62, and 65, respectively; Fig. 1B; Supplementary Table S2).

Interestingly, Group 3 had the highest mTMB compared with Groups 1 and 2, across the three cancer types (*P* < 0.001 for colorectal cancer and endometrial cancer, *P* < 0.05 for ovarian cancer; Fig. 1C–E; Supplementary Table S3), even when MSI-H tumors were excluded from the analysis [significant differences (*P* < 0.001) for colorectal cancer and endometrial cancer, but not for ovarian cancer, likely due to small sample size; Fig. 1F–H; Supplementary Table S3]. We validated our findings by analyzing the sequencing data from TCGA, which included 46 tumors (78% endometrial cancer or colorectal cancer) with *POLE* variants (access date: February 2022). In this dataset, a significantly higher mTMB was observed in Group 3 (*POLE* ExoD driver plus *POLE* Variant) versus Group 2 (*POLE* ExoD driver; *P* < 0.001; Fig. 1I; Supplementary Table S4), even when excluding MSI-H tumors from the analysis (*P* < 0.001; Fig. 1J; Supplementary Table S4).

**FIGURE 2:**
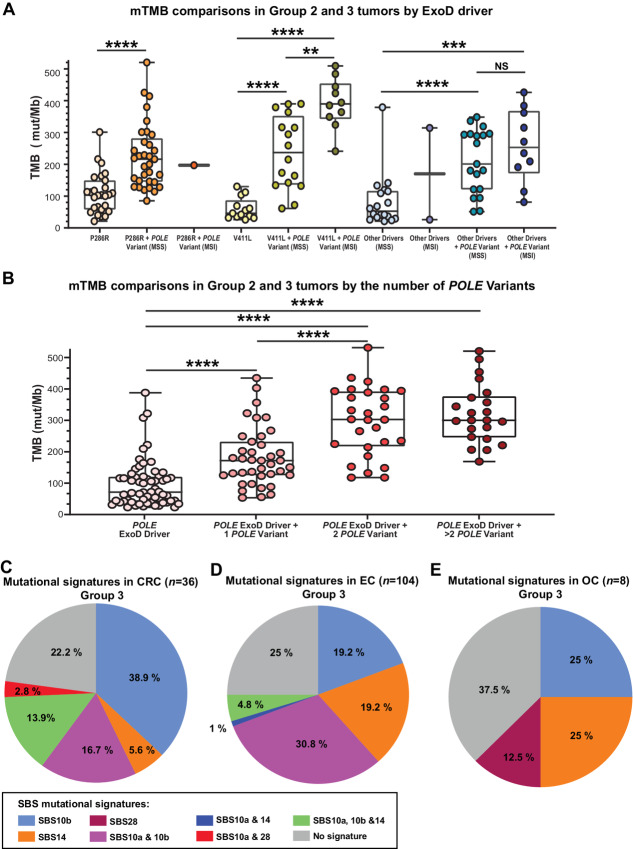
**A,** mTMB comparisons in Group 2 and 3 tumors by ExoD driver alone [P286R, V411L, or other driver(s) combined] or in conjunction with *POLE* variants. Each filled round circle represents a tumor genomic profile; data are shown for the driver alone and/or plus *POLE* variant. Data in “other drivers” were combined because of lower numbers. The data are segregated for MSS or MSI status where relevant. A few statistical comparisons were not performed because of ≤2 datapoints. **B,** mTMB comparisons in Group 2 and 3 tumors by the increasing number of *POLE* variants. Data are combined analysis of colorectal cancer, endometrial cancer, and ovarian cancer genomic profiles in the Caris dataset. Each filled round circle represents a tumor genomic profile; data are shown for Group 2 ExoD drivers, and for Group 3 by the ExoD driver plus the number of variants [1, 2, and >2 variant(s)]. A and B, A Mann–Whitney test was performed and ****, *P* < 0.0001; ***, *P* < 0.001; **, *P* < 0.01; NS, nonsignificant. Corrections for multiple comparisons were performed using the Benjamini–Hochberg FDR test. **C**–**E,** 3-nucleotide context of *POLE* variants in Group 3 tumors. All COSMIC mutational signatures associated with POLE ExoD driver defects (SBS 10a, SBS 10b, SBS 14, and SBS 28) were assessed (39). Pie chart distribution of SBS 10a, SBS 10b, SBS 14, and SBS 28 in colorectal cancer (**C**), endometrial cancer (**D**), and ovarian cancer (E). Each of these signatures has a primary mutation which has been described as a “hotspot” (39); SBS 10a is C>A in TCT context; SBS 10b is C>T in the TCG context; SBS 14 is C>A in the NCT context (N is any base); and SBS 28 is T>G in the TTT context. In addition to these primary “hotspots,” all mutations that comprise >1% of the genome signature of interest were counted, capturing 88%–90% of each signature in the analysis.

Because of the consistent findings in the CLS dataset across all three cancer types, we merged them to enhance the statistical power. As expected previously (39), P286R and V411 L were the two most common *POLE* ExoD drivers across the three cancer types in the CLS dataset, present in 67% of tumors with a *POLE* ExoD driver. Analyzing tumors by specific POLE ExoD driver (P286R, V411 L, or any other ExoD driver) confirmed the independent nature of the association, that is, increased mTMB with addition of *POLE* variants is independent of the POLE ExoD driver (Fig. 2A). When assessing how TMB changed with an increasing number of *POLE* variants in the presence of a POLE ExoD driver (Fig. 2B), mTMB significantly, and progressively, increased as the number of *POLE* variants increased from 0 to 2; however, it plateaued after acquiring two *POLE* variants (Fig. 2B, *P* < 0.001). This indicates that the association between the increasing mTMB and the additional *POLE* variant(s) in Group 3 tumors may not solely reflect increased mutagenesis.

### Location and Nature of the *POLE* Variants Identified in Group 3 Tumors

The *POLE* variants that accompanied the ExoD drivers (Group 3) included: 12 ExoD variants (all missense), and 143 non-ExoD variants [12 disruptive (2 frameshift and 10 nonsense), and 131 missense]. Group 3 tumors had COSMIC mutation signatures SBS 10a/10b as would be expected with the presence of an established *POLE* ExoD driver (Supplementary Fig. S1). To investigate whether *POLE* variants in Group 3 tumors resulted from increased proofreading defects linked to the POLE ExoD driver, we analyzed the 3-nucleotide context of *POLE* variants in Group 3 tumors. Park and colleagues employed the 3-nucleotide context approach to demonstrate that, in the presence of a POLE ExoD driver, tumor mutations predominantly emerge within certain nucleotide trios (40). These trios have been linked to COSMIC mutational signatures SBS 10a, 10b, 14, and 28, all of which are indicative of a POLE proofreading defect (39–41). Thus, we assessed each *POLE* variant in Group 3 tumors, according to the cancer type and overall, considering the 3-nucleotide context associated with COSMIC mutational signatures SBS 10a, 10b, 14, and 28 [Fig. 2C–2E; Supplementary Fig. S2A (overall)]. In colorectal cancer, the primary mutation associated with SBS 10b (TCG>TTG) comprised 36.1% of *POLE* variants. TCT>TAT is considered primary for both SBS 10a and SBS 14 and comprised 13.9% of *POLE* variants (Fig. 2C). Overall, in colorectal cancer, 77.8% of the *POLE* variants occurred in *POLE* ExoD signature sequence contexts (Fig. 2C). In endometrial cancer, TCG>TTG substitutions comprised 17.3% of *POLE* variants, and TCT>TAT comprised 4.8% of variant *POLE* variants. Interestingly, GCG>GTG, which is a minor mutation associated with both the SBS 10a and SBS 10b signatures, comprised 26.9% of *POLE* variants. This specific mutation has been previously associated with *POLE* driver mutations with defective MMR (39). CCG>CTG, which is a minor mutation in SBS 14, was also a common mutation in Group 3 tumors (10.6% of *POLE* variants). Overall, in endometrial cancer, 75% of the *POLE* variants occurred in *POLE* ExoD signature sequence contexts (Fig. 2D). In ovarian cancer, there were two TCG>TTG mutations of SBS 10b and two minor mutations of SBS 14. The ovarian cancer group has a small sample size: of these, 62.5% of the *POLE* variants occurred in *POLE* ExoD signature sequence contexts (Fig. 2E). In summary, the analysis of the selected COSMIC mutational signatures provides compelling evidence that most *POLE* variants within Group 3 tumors are within the distinct mutation context of the POLE proofreading defect.

### *POLE* Variants in MSS TMB-H Subset of Group 4

In Group 4 tumors, the *POLE* variants were: 27 ExoD variants (two deletion-insertion, one splice site variant, and 24 missense variants) and 127 non-ExoD variants (eight nonsense mutations, 10 frameshift mutations, five deletions, one duplication, one insertion, two canonical splice site variants, and 100 missense variants; Supplementary Fig. S2B; Supplementary Table S1). When segregating by cancer type, *POLE* variants in Group 4 were observed in colorectal cancer (*n* = 21), endometrial cancer (*n* = 118), and in ovarian cancer (*n* = 4). Among the MSS TMB-H subset of Group 4 tumors, *POLE* variants associated with TMB-H were observed in colorectal cancer and endometrial cancer (*n* = 14); most variants were missense (8/14), but nonsense and other alterations were also observed (6/14). Here, six variants were in the CTD (E1376D, R1386W, Q1475X, P1547S, Y1889X, S1930X) and two variants were in the polymerase domain (R680C, palm; R1125X, thumb). ExoD variants (3/14) were observed as missense [M295R (in colorectal cancer and endometrial cancer)], F320V, M444I (also reported in ref. 44), or as chromosomal alterations (F285_P286delinsLR and N423_L424delinsKI). We speculate that the *POLE* variants in MSS tumors with TMB-H could be potential new drivers; requiring functional validation.

### Molecular Features of *POLE*-mutated Tumor Groups

**FIGURE 3:**
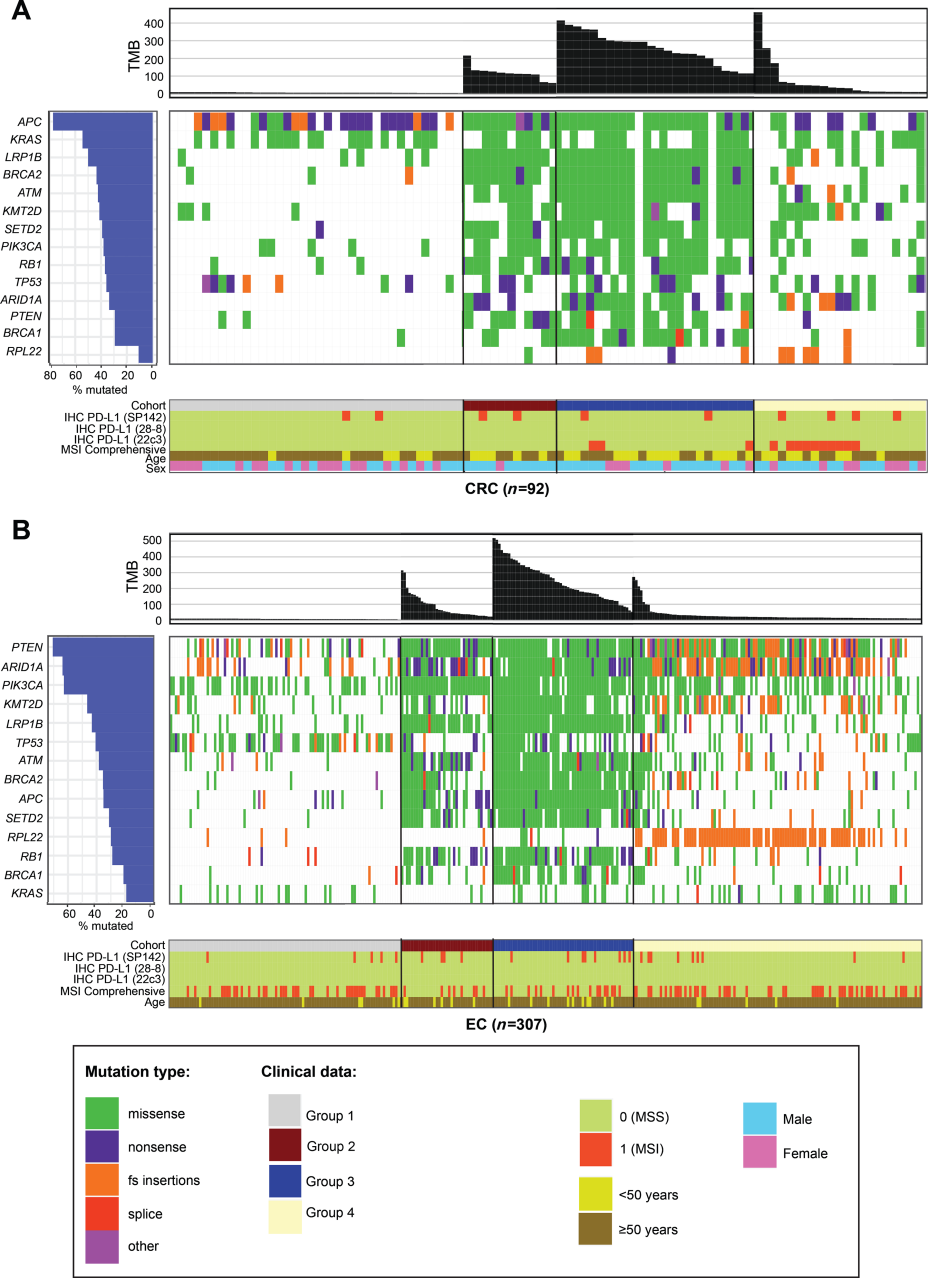
Molecular features of colorectal cancer (**A**) and endometrial cancer (**B**). The mutational landscape and patient demographic/clinical characteristics (PD-L1 by IHC, MSI comprehensive, age, and sex) of the four cohorts for each cancer type were plotted using the GenVisR package of R.

To assess whether the presence of the non-driver *POLE* variants was reflected in the nature of observed oncogenic driver mutations, we analyzed the molecular features of each individual group per cancer type in the CLS dataset (Fig. 3, colorectal cancer, and endometrial cancer; Supplementary Fig. S3; ovarian cancer; Supplementary Table S1 for all the data in the figures). The data show comutated genes either unique to and/or more prevalent in Groups 2 and 3 tumors, such as *LRP1B, ATM, BRCA1, BRCA2, SETD2, ARID1A,* and *KMT2D*.

In colorectal cancer, *APC* was the most common comutated gene as expected; however, Groups 1 and 4 *APC* mutations were typically nonsense and frameshift while Groups 2 and 3 mutations were missense (Fig. 3A; Supplementary Table S1). In *KRAS*-mutated colorectal cancer, the most frequent mutations in Group 1 were KRAS G12V or G12D, whereas in Group 4, most common were KRAS G13D or G12D (Supplementary Table S1). In contrast, KRAS A146T—overall, a less commonly observed KRAS mutation (45)—was the most frequent KRAS mutation in Groups 2 and 3, and not found in Groups 1 and 4. The nucleotide context of the KRAS A146T is a POLE mutation signature (Supplementary Table S1).

In endometrial cancer, *ARID1A, PTEN*, and *PIK3CA* were commonly mutated across all groups (Fig. 3B). *ARID1A* mutations in Groups 1 and 4 were frameshift, typically missense in Group 3, and a mix of missense, nonsense, and frameshift in Group 2. The preference for the PTEN R130 hotspot inactivating mutation (R130*, R130G/Q; refs. 46–49) differed between the groups (Supplementary Table S1); PTEN R130Q was most frequent in Groups 2 and 3, and its nucleotide context is a POLE mutation signature (40). Other relevant PTEN mutations either unique or more frequent in Groups 2 and 3 were E7*, R15K, E299*/X, R142W, F154L/V, R173C, F341V/C; some of these have been previously observed in *POLE* MSS high TMB colorectal cancers (49) and their nucleotide context is a POLE mutation signature (ref. 40; Supplementary Table S1). Frameshift *PTEN* mutations were not found in Groups 2 and 3. In PIK3CA-mutated endometrial cancers, preference for the type of activating gain-of-function mutation (R88Q, E452K, E454K, H1047R, M1043I; ref. 50) differed between the groups; PIK3CA R88Q was most frequent in Groups 2 and 3, and its nucleotide context is a POLE mutation signature (Supplementary Table S1).

Neoantigen prediction in Group 2 and 3 tumors for colorectal and endometrial tumors demonstrated that the abundance of tumor neoantigens was significantly greater in Group 3 tumors (Supplementary Fig. S4; Supplementary Table S5). Finally, in all cancers studied, we did not observe any correlation between PD-L1 expression and presence of a *POLE* ExoD driver (Fig. 3; Supplementary Fig. S3; Supplementary Table S1). Together, these data highlight the unique landscape of *POLE* ExoD-mutated Groups 2 and 3 tumors. Importantly, no additional genetic factors that differ between these groups were identified.

### POLE Missense Variants in Groups 2, 3, and 4 Tumors; Association with Protein Stability

**FIGURE 4:**
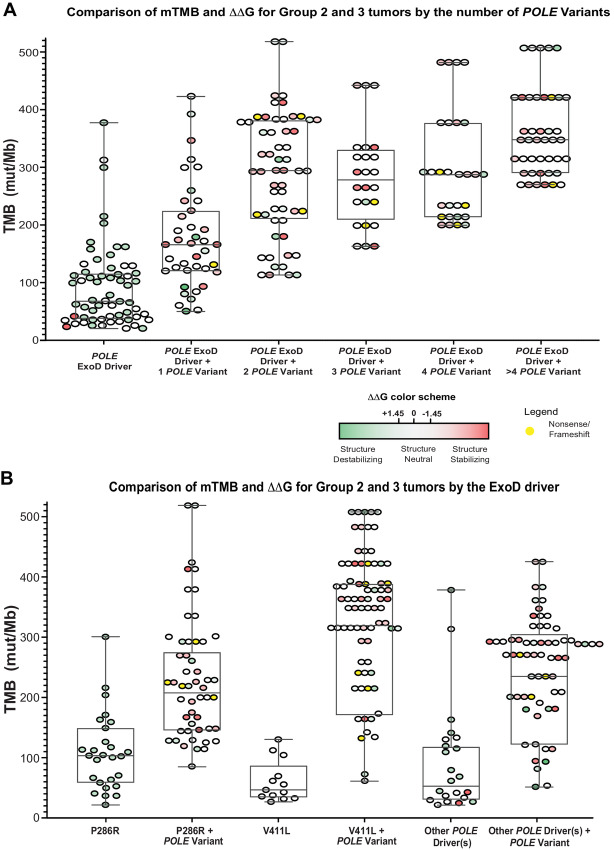
Comparison of mTMB and ΔΔG values in Group 2 and 3 tumors in the CLS dataset. With AF2 DNA unbound model and Rosetta ddG_monomer, we generated 25 repacked decoys for each mutation and compared the average energy score for these decoys with an average for 25 decoys of the WT protein. For mutations in the NTL, we performed calculations on both models (with and without DN). For those in the CTL, we performed calculations only on the DNA unbound model. We used a cutoff of ±1.45 kcal/mol for significant ΔΔG, corresponding to approximately 3 SDs of the differences of the mean Rosetta scores for WT and mutant structures. **A,** Comparison of mTMB and ΔΔG values in Group 2 and Group 3 tumors by the number of *POLE* variants. Data for colorectal cancer, endometrial cancer, and ovarian cancer genomic profiles were combined, and ΔΔG values were plotted against the mTMB. For Group 2 or 3 data with + 1 *POLE* variant plots, each filled round circle represents a single tumor genomic profile. **B,** Comparison of mTMB and ΔΔG values in Group 2 and Group 3 tumors by *POLE* ExoD driver. Data for colorectal cancer, endometrial cancer, and ovarian cancer genomic profiles were combined, and ΔΔG values were plotted against the mTMB. A and B, Group 3 tumors with multiple variants, a circle next to another circle (without any space) represents a single tumor. For clarity, ΔΔG values for ExoD drivers in Group 3 tumors are not shown (they are same as in Group 2). Color in each filled circle—green and shades of green, structure-destabilizing variants (positive ΔΔG); white, variants that are within the SDs of ±1.45 kcal/mol and are structure neutral; red and shades of red, structure-stabilizing variants (negative ΔΔG). Yellow, nonsense, or frameshift variants.

The values for the WT AF2 structure models are highly reproducible: for instance, the SD of the mean energies for 5 runs without DNA was only 0.16 kcal/mol. Using a cutoff of 1.45 kcal/mol for significant ΔΔG, Group 2 and 3 tumors were annotated by mTMB and the number of *POLE* variants (Fig. 4A, DNA unbound model; see Supplementary Fig. S5A, DNA bound model). Group 2 tumors contained mostly destabilizing ExoD driver mutations according to Rosetta ddG_monomer (Fig. 4A, ΔΔG ≥ +1.45 kcal/mol). In contrast, the POLE variants in Group 3 tumors are mostly stabilizing (Fig. 4A, ΔΔG ≤ −1.45 kcal/mol). Furthermore, Group 3 tumors with a P286R driver generally had one or two additional POLE variants, where most are structure stabilizing (Fig. 4B; see Supplementary Fig. S5B, DNA bound model). Group 3 tumors with V411 L driver that had the highest mTMBs tended to have multiple POLE variants (2 to 8) per tumor, with a range of structure stabilizing or destabilizing variants. For Group 3 tumors with other drivers, most POLE variants were structure stabilizing.

To further analyze POLE missense variants in Groups 2, 3, and 4, the AF2 POLE structure models were used to annotate and analyze each variant by the domain (Supplementary Fig. S6A and S6B; Supplementary Fig. S2B; *n* = 168 variants). A total of 35 destabilizing (ΔΔG ≥ +1.45 kcal/mol) and 17 stabilizing (ΔΔG ≤ −1.45 kcal/mol) missense variants were located at the N-terminal lobe (NTL) in the DNA-unbound model. Overall, we found that structure-destabilizing missense variants were more prevalent in the NTL than the C-terminal lobe (CTL) (see Supplementary Table S6; red = stabilizing; green = destabilizing). The 20 ExoD driver residues (Supplementary Fig. S6C; Supplementary Table S6) are almost all buried in the hydrophobic core of the ExoD, even though they are not all hydrophobic. The most destabilizing driver mutations in both the without-DNA (all >4.0 kcal/mol) and with-DNA (all >2.8 kcal/mol) are of hydrophobic amino acids: P286R, L424F, P436S, P436Y, P436R, M444K, A456P. Other structure destabilizing driver mutations of hydrophobic amino acids are F367C, S459Y, and S461P. These drivers are almost all either in the central helix of the ExoD [“Exo III motif” (51)] or in contact with it (Supplementary Fig. S6C); implying an effect on stability or dynamics of the ExoD.

In Group 3 tumors with P286R driver, all but one of the 20 mutations at 17 sites have TMB above the median value for P286R driver alone (mTMB = 103; Supplementary Table S7; Supplementary Fig. S6D and S6E). In Group 3 tumors with V411 L driver, there are seven tumors with V411 L + one variant, and they all have TMB above the median value of V411 L driver alone (mTMB = 44; Supplementary Fig. S6D and S6F; Supplementary Table S7). Supplementary Figure S6G shows POLE variants from the NTD subdomain and the ExoD domain with striking ΔΔG values, some with the most highly destabilizing ΔΔG values. Overall, future biochemical and cellular studies will support the impact of these variants.

## Discussion

It is currently not known why tumors of a given cancer type and with the same *POLE* ExoD driver have different levels of TMB, that is, some show hypermutation (10–100 mut/Mb) while others are ultra-hypermutated (>100 mut/Mb). This study uncovers a distinct subset of highly mutated *POLE* ExoD–mutated tumors with additional *POLE* variants in several folded domains of POLE. Our findings indicate, at least in a subset of tumors, ultra-hypermutation may be associated with the acquisition of one or more additional variants in *POLE*, beyond the established ExoD driver. In fact, the 3-nucleotide context of these *POLE* variants suggests that they are secondary to the ExoD proofreading defect in those tumors. In the three cancer types studied, colorectal cancer, endometrial cancer, and ovarian cancer, TMB was significantly higher when the corresponding *POLE* ExoD driver mutation was present in conjunction with one or more additional *POLE* variant(s). Notably, this association remained significant in colorectal cancer and endometrial cancer tumors even after MSI-H tumors were excluded from the analysis (but not for ovarian cancer, likely due to the smaller sample size). Our findings were further validated in polymerase ε proofreading–deficient tumors from TCGA. The results shown also suggest that the observed increase in mTMB is not solely due to increased mutagenesis but rather implicate a modifier role for the additional *POLE* variants. As observed before (12), tumors with *POLE* ExoD driver mutations were diagnosed earlier than proofreading proficient tumors without a *POLE* ExoD driver. Moreover, we also observed a trend to earlier age of onset for colorectal cancers and endometrial cancers with *POLE* ExoD driver occurring in conjunction with other *POLE* variants.

Restricting TMB analysis to specific ExoD hotspot drivers showed that both stronger (e.g., P286R) and weaker (e.g., V411L) ExoD drivers had additional POLE variant(s). However, tumors with V411 L or other drivers had more *POLE* variants versus tumors with P286R, which at most harbored one additional POLE variant. Recent studies have revealed several differences in proofreading-defective *POLE*-mutated tumors. lt has been shown that all ExoD mutations do not have strong mutagenic effects, and sometimes mutations in the polymerase domain can be associated with hypermutation (10, 39). Typically, patient tumor genomic profiles and tumor cell lines do not exhibit evidence of LOH for the *POLE* ExoD driver mutation (52). Our data suggest that while inactivation of exonuclease activity is sufficient to drive mutagenesis, it may not always be sufficient to drive ultra-hypermutagenesis, especially in the case of ExoD drivers with weaker mutagenic effect (e.g., V411L). The 3-nucleotide sequence contexts of the *POLE* variants in Group 3 tumors strongly suggest that the *POLE* ExoD driver mutation first made the DNA synthesis error, and the cells with the additional *POLE* variant subsequently proliferated and expanded during tumor development. While most additional *POLE* variants were secondary, a minor subset did not fall under the signature sequence contexts associated with POLE defects and could be pre-existing; limitations of retrospective data prevent further analysis on when these mutations emerged. Finally, we found five tumors that carried two known ExoD drivers as opposed to a single ExoD driver; limitations of retrospective data prevent further analysis on which ExoD driver was acquired first.

The sheer number of mutations in proofreading-defective tumors makes it difficult to identify mutations that have a functional impact. Our analysis found enriched *POLE* variants and several oncogenic driver mutations (e.g., KRAS A146T) occur in the POLE proofreading defect mutation signature contexts. In addition, the comutation analysis also emphasizes the unique mutational profile of *POLE-*driven Groups 2 and 3 tumors. Mutations in *LRP1B*, *ARID1A*, *KMT2D,* and *SETD2* have been associated independently as biomarkers of better outcome from checkpoint blockade immunotherapy (53–56). Moreover, comutations in *BRCA1* or *BRCA2* suggest the potential use of PARP inhibitor therapy for Groups 2 and 3 tumors. Furthermore, findings of mutations in genes such as *BRCA* genes, *ATM, SETD2, ARID1A* involved in DNA damage surveillance, and chromatin modification provide evidence to support the notion that the pathogenesis of *POLE*-driven tumors involves a compromised DNA damage response or repair mechanism (40). *APC* mutations in colorectal cancer are typically truncating (57–59). The missense *APC* mutations, found exclusively in Groups 2 and 3, have been reported to alter protein activity in a more subtle manner through the expression of differentially spliced forms of APC (57). The results consistently indicated that there are no other substantial genetic factors differing between Group 2 and 3 tumors to account for the increased mutagenicity observed in Group 3 tumors. This study offers intriguing insights into the abundance of neoantigens in Group 3 tumors, though this aspect warrants deeper exploration. For a comprehensive understanding of potential clinical impacts, subsequent analyses of immunogenic epitopes should consider a wider range of pertinent features, as thoroughly discussed in ref. 60. Moving forward, research should focus on establishing the clinical significance of our findings to refine therapeutic approaches.

Previously, only simple homology models of human POLE from yeast POLE structures have been used in the structure-function analysis of POLE mutations (11, 61). The use of AF2 in this study to model human POLE more accurately allowed us to calculate protein stability changes in the mutant versus the WT proteins to provide unique insights into the known ExoD drivers and POLE variants. Protein stability can more accurately predict changes due to mutations especially missense mutations in proteins (62). We found that while most established ExoD drivers are predicted to be structure destabilizing, there are few established drivers that are either predicted to be structure stabilizing or have no significant predicted impact on stability. For the POLE variants that co-occurred with the ExoD driver, the impact on stability differed by regions or domains; more structure-destabilizing variants were more prevalent in the NTL compared with the CTL. It is possible to speculate that variants in certain regions/domains of POLE (occurring in conjunction with the driver) may lead to other mechanisms increasing mutagenesis beyond simple defects in proofreading. In fact, these other mechanisms beyond simple proofreading defect have already begun to be associated with driver mutations; for example, the P286R mutation is thought to produce a hyperactive DNA polymerization state which amplifies the proofreading defect (63).

Our study is hypothesis generating and nominates several questions that need to be addressed in future studies using biochemical activity assays and/or cellular models. These future studies should aim to investigate the secondary *POLE* variants described here in an isogenic genetic background with and without an ExoD driver allele. This approach would provide direct evidence regarding whether the secondary POLE variants are a cause or a consequence of high mutation rate. Moreover, the data in this study may have implications for clinical management of patients with *POLE*-mutated tumors. It is important to understand whether there is a systematic clinical benefit associated with tumors carrying specific ExoD drivers plus additional variant(s). Recent studies suggest that this may indeed be the case (11–13). Garmezy and colleagues recently reported better clinical outcomes in patients with *POLE* pathogenic variants and in patients with *POLE* VUS (12). They showed that most VUS that correlated with better outcomes affected other regions of POLE apart from the ExoD (12). Rousseau and colleagues found that individuals with tumors bearing *POLE* VUS within the ExoD catalytic site, or the DNA binding site showed clinical benefit from nivolumab (11). Another study observed that high TMBs (median: 275.38/Mb) in tumors with POLE proofreading and MMR deficiency were significantly associated both with response to ICIs and survival (13). This suggests not all tumors with high TMB may exhibit the same clinical benefit, and that analysis by specific TMB thresholds (which would largely include Group 3 tumors identified in this study) may demonstrate better outcomes. Further investigation is needed to fully elucidate the clinical implications of the findings presented here. In the meantime, only select pathogenic *POLE* mutations in the ExoD, linked to ultra-mutation, have shown clinical benefit. In the absence of a pathogenic POLE ExoD mutation, the presence of a POLE non-ExoD mutation should be considered a VUS and not alter clinical practice.

This study has several limitations: the data are retrospective, and the analysis presented here cannot fully capture the impact of *POLE* variants in conjunction with *POLE* ExoD drivers based on limitations in the dataset on response to therapy, exposures, ancestry/ethnicity. The study cannot always exclude the variants analyzed as germline versus somatic. In addition, larger sample sizes and longer clinical follow-up studies are needed to investigate the long-term outcomes of patients with such tumors. Overall, these data support future mechanistic studies on the synergy between additional *POLE* variants and *POLE* ExoD driver mutations. This could be pivotal in comprehending not just tumor development but also potential impact on clinical outcomes.

## Supplementary Material

Supplementary Data All Supplementary_Legends_Figures_and_most Tables

Supplementary Table 1 All patient clinical, demographic, and molecular data in this manuscript (de-identified).

Supplementary Table 2 Age Distribution of CRC, EC, and OC patients with POLE-mutated tumors.

Supplementary Table 3 mTMB comparisons in the Caris Life Sciences dataset.

Supplementary Table 4 mTMB comparisons in TCGA dataset.

Supplementary Table 5 Neoantigens predictions for Group 3 tumor mutations by DeepNeo (sheet 1 for CRC; sheet 2 for EC) and Neodb (sheet 3 for CRC, and EC).

Supplementary Table 6 Rosetta ΔΔG values for POLE variants and drivers with or without DNA

Supplementary Table 7 Mutations in Group 3 tumors with P286R or V411L plus one variant and mTMB comparisons.

## References

[1] Nicolas E , Golemis EA, Arora S. POLD1: central mediator of DNA replication and repair, and implication in cancer and other pathologies. Gene 2016;590:128–41. 27320729 10.1016/j.gene.2016.06.031 PMC4969162

[2] Vande Perre P , Siegfried A, Corsini C, Bonnet D, Toulas C, Hamzaoui N, . Germline mutation p.N363K in POLE is associated with an increased risk of colorectal cancer and giant cell glioblastoma. Fam Cancer 2019;18:173–8. 30368636 10.1007/s10689-018-0102-6

[3] Mur P , García-Mulero S, Del Valle J, Magraner-Pardo L, Vidal A, Pineda M, . Role of POLE and POLD1 in familial cancer. Genet Med 2020;22:2089–100. 32792570 10.1038/s41436-020-0922-2 PMC7708298

[4] Palles C , Cazier JB, Howarth KM, Domingo E, Jones AM, Broderick P, . Germline mutations affecting the proofreading domains of POLE and POLD1 predispose to colorectal adenomas and carcinomas. Nat Genet 2013;45:136–44. 23263490 10.1038/ng.2503 PMC3785128

[5] Valle L , Hernández-Illán E, Bellido F, Aiza G, Castillejo A, Castillejo M-I, . New insights into POLE and POLD1 germline mutations in familial colorectal cancer and polyposis. Hum Mol Genet 2014;23:3506–12. 24501277 10.1093/hmg/ddu058

[6] Djursby M , Madsen MB, Frederiksen JH, Berchtold LA, Therkildsen C, Willemoe GL, . New pathogenic germline variants in very early onset and familial colorectal cancer patients. Front Genet 2020;11:566266. 33193653 10.3389/fgene.2020.566266 PMC7541943

[7] Germline DNA polymerase mutations increase cancer susceptibility. Cancer Discov 2013;3:136.

[8] Ahn S-M , Ahmad Ansari A, Kim J, Kim D, Chun S-M, Kim J, . The somatic POLE P286R mutation defines a unique subclass of colorectal cancer featuring hypermutation, representing a potential genomic biomarker for immunotherapy. Oncotarget 2016;7:68638–49. 27612425 10.18632/oncotarget.11862 PMC5356579

[9] León-Castillo A , Britton H, McConechy MK, McAlpine JN, Nout R, Kommoss S, . Interpretation of somatic POLE mutations in endometrial carcinoma. J Pathol 2020;250:323–35. 31829442 10.1002/path.5372 PMC7065171

[10] Campbell BB , Light N, Fabrizio D, Zatzman M, Fuligni F, de Borja R, . Comprehensive analysis of hypermutation in human cancer. Cell 2017;171:1042–56. 29056344 10.1016/j.cell.2017.09.048 PMC5849393

[11] Rousseau B , Bieche I, Pasmant E, Hamzaoui N, Leulliot N, Michon L, . PD-1 blockade in solid tumors with defects in polymerase epsilon. Cancer Discov 2022;12:1435–48. 35398880 10.1158/2159-8290.CD-21-0521 PMC9167784

[12] Garmezy B , Gheeya J, Lin HY, Huang Y, Kim T, Jiang X, . Clinical and molecular characterization of pole mutations as predictive biomarkers of response to immune checkpoint inhibitors in advanced cancers. JCO Precis Oncol 2022;6:e2100267. 35108036 10.1200/PO.21.00267 PMC8820927

[13] Das A , Sudhaman S, Morgenstern D, Coblentz A, Chung J, Stone SC, . Genomic predictors of response to PD-1 inhibition in children with germline DNA replication repair deficiency. Nat Med 2022;28:125–35. 34992263 10.1038/s41591-021-01581-6 PMC8799468

[14] Chung J , Maruvka YE, Sudhaman S, Kelly J, Haradhvala NJ, Bianchi V, . DNA polymerase and mismatch repair exert distinct microsatellite instability signatures in normal and malignant human cells. Cancer Discov 2021;11:1176–91. 33355208 10.1158/2159-8290.CD-20-0790 PMC8223607

[15] Rahn S , Krüger S, Mennrich R, Goebel L, Wesch D, Oberg HH, . POLE score: a comprehensive profiling of programmed death 1 ligand 1 expression in pancreatic ductal adenocarcinoma. Oncotarget 2019;10:1572–88. 30899426 10.18632/oncotarget.26705 PMC6422186

[16] Haruma T , Nagasaka T, Nakamura K, Haraga J, Nyuya A, Nishida T, . Clinical impact of endometrial cancer stratified by genetic mutational profiles, POLE mutation, and microsatellite instability. PLoS One 2018;13:e0195655. 29659608 10.1371/journal.pone.0195655 PMC5901772

[17] Merino DM , McShane LM, Fabrizio D, Funari V, Chen SJ, White JR, . Establishing guidelines to harmonize tumor mutational burden (TMB): in silico assessment of variation in TMB quantification across diagnostic platforms: phase I of the Friends of Cancer Research TMB Harmonization Project. J Immunother Cancer 2020;8:e000147. 32217756 10.1136/jitc-2019-000147 PMC7174078

[18] Marabelle A , Fakih M, Lopez J, Shah M, Shapira-Frommer R, Nakagawa K, . Association of tumour mutational burden with outcomes in patients with advanced solid tumours treated with pembrolizumab: prospective biomarker analysis of the multicohort, open-label, phase 2 KEYNOTE-158 study. Lancet Oncol 2020;21:1353–65. 32919526 10.1016/S1470-2045(20)30445-9

[19] Bartley AN , Mills AM, Konnick E, Overman M, Ventura CB, Souter L, . Mismatch repair and microsatellite instability testing for immune checkpoint inhibitor therapy: guideline from the College of American Pathologists in collaboration with the association for molecular pathology and fight colorectal cancer. Arch Pathol Lab Med 2022;146:1194–210. 35920830 10.5858/arpa.2021-0632-CP

[20] Fortuno C , Lee K, Olivier M, Pesaran T, Mai PL, de Andrade KC, . Specifications of the ACMG/AMP variant interpretation guidelines for germline TP53 variants. Hum Mutat 2021;42:223–36. 33300245 10.1002/humu.24152 PMC8374922

[21] Skidmore ZL , Wagner AH, Lesurf R, Campbell KM, Kunisaki J, Griffith OL, . GenVisR: Genomic Visualizations in R. Bioinformatics 2016;32:3012–4. 27288499 10.1093/bioinformatics/btw325 PMC5039916

[22] Cerami E , Gao J, Dogrusoz U, Gross BE, Sumer SO, Aksoy BA, . The cBio cancer genomics portal: an open platform for exploring multidimensional cancer genomics data. Cancer Discov 2012;2:401–4. 22588877 10.1158/2159-8290.CD-12-0095 PMC3956037

[23] Gao J , Aksoy BA, Dogrusoz U, Dresdner G, Gross B, Sumer SO, . Integrative analysis of complex cancer genomics and clinical profiles using the cBioPortal. Sci Signal 2013;6:pl1. 23550210 10.1126/scisignal.2004088 PMC4160307

[24] Díaz-Gay M , Vila-Casadesús M, Franch-Expósito S, Hernández-Illán E, Lozano JJ, Castellví-Bel S. Mutational signatures in cancer (MuSiCa): a web application to implement mutational signatures analysis in cancer samples. BMC Bioinformatics 2018;19:224. 29898651 10.1186/s12859-018-2234-y PMC6001047

[25] Demidova EV , Serebriiskii IG, Vlasenkova R, Kelow S, Andrake MD, Hartman TR, . Candidate variants in DNA replication and repair genes in early-onset renal cell carcinoma patients referred for germline testing. BMC Genomics 2023;24:212. 10.1186/s12864-023-09310-8 PMC10123997 37095444

[26] Hogg M , Osterman P, Bylund GO, Ganai RA, Lundström EB, Sauer-Eriksson AE, . Structural basis for processive DNA synthesis by yeast DNA polymerase ɛ. Nat Struct Mol Biol 2014;21:49–55. 24292646 10.1038/nsmb.2712

[27] Yuan Z , Georgescu R, Schauer GD, O'Donnell ME, Li H. Structure of the polymerase ε holoenzyme and atomic model of the leading strand replisome. Nat Commun 2020;11:3156. 32572031 10.1038/s41467-020-16910-5 PMC7308368

[28] Alford RF , Leaver-Fay A, Jeliazkov JR, O'Meara MJ, DiMaio FP, Park H, . The rosetta all-atom energy function for macromolecular modeling and design. J Chem Theory Comput 2017;13:3031–48. 28430426 10.1021/acs.jctc.7b00125 PMC5717763

[29] Khatib F , Cooper S, Tyka MD, Xu K, Makedon I, Popovic Z, . Algorithm discovery by protein folding game players. Proc Natl Acad Sci U S A 2011;108:18949–53. 22065763 10.1073/pnas.1115898108 PMC3223433

[30] Maguire JB , Haddox HK, Strickland D, Halabiya SF, Coventry B, Griffin JR, . Perturbing the energy landscape for improved packing during computational protein design. Proteins 2021;89:436–49. 33249652 10.1002/prot.26030 PMC8299543

[31] Krivov GG , Shapovalov MV, Dunbrack RL Jr. Improved prediction of protein side-chain conformations with SCWRL4. Proteins 2009;77:778–95. 19603484 10.1002/prot.22488 PMC2885146

[32] Shapovalov MV , Dunbrack RL Jr. A smoothed backbone-dependent rotamer library for proteins derived from adaptive kernel density estimates and regressions. Structure 2011;19:844–58. 21645855 10.1016/j.str.2011.03.019 PMC3118414

[33] Pettersen EF , Goddard TD, Huang CC, Couch GS, Greenblatt DM, Meng EC, . UCSF Chimera–a visualization system for exploratory research and analysis. J Comput Chem 2004;25:1605–12. 15264254 10.1002/jcc.20084

[34] Jumper J , Evans R, Pritzel A, Green T, Figurnov M, Ronneberger O, . Highly accurate protein structure prediction with AlphaFold. Nature 2021;596:583–9. 34265844 10.1038/s41586-021-03819-2 PMC8371605

[35] Tunyasuvunakool K , Adler J, Wu Z, Green T, Zielinski M, Žídek A, . Highly accurate protein structure prediction for the human proteome. Nature 2021;596:590–6. 34293799 10.1038/s41586-021-03828-1 PMC8387240

[36] Leaver-Fay A , O'Meara MJ, Tyka M, Jacak R, Song Y, Kellogg EH, . Scientific benchmarks for guiding macromolecular energy function improvement. Methods Enzymol 2013;523:109–43. 23422428 10.1016/B978-0-12-394292-0.00006-0 PMC3724755

[37] Shah SM , Demidova EV, Ringenbach S, Faezov B, Andrake M, Mur P, . Co-occurring mutations in the POLE exonuclease and non-exonuclease domains define a unique subset of highly mutagenic tumors. bioRxiv 2022.

[38] Alexandrov LB , Kim J, Haradhvala NJ, Huang MN, Tian Ng AW, Wu Y, . The repertoire of mutational signatures in human cancer. Nature 2020;578:94–101. 32025018 10.1038/s41586-020-1943-3 PMC7054213

[39] Hodel KP , Sun MJS, Ungerleider N, Park VS, Williams LG, Bauer DL, . POLE mutation spectra are shaped by the mutant allele identity, its abundance, and mismatch repair status. Mol Cell 2020;78:1166–77. 32497495 10.1016/j.molcel.2020.05.012 PMC8177757

[40] Park VS , Sun MJS, Frey WD, Williams LG, Hodel KP, Strauss JD, . Mouse model and human patient data reveal critical roles for Pten and p53 in suppressing POLE mutant tumor development. NAR Cancer 2022;4:zcac004. 35252866 10.1093/narcan/zcac004 PMC8892059

[41] Robinson PS , Coorens THH, Palles C, Mitchell E, Abascal F, Olafsson S, . Increased somatic mutation burdens in normal human cells due to defective DNA polymerases. Nat Genet 2021;53:1434–42. 34594041 10.1038/s41588-021-00930-y PMC8492474

[42] Kim JY , Bang H, Noh SJ, Choi JK. DeepNeo: a webserver for predicting immunogenic neoantigens. Nucleic Acids Res 2023;51:W134–40. 37070174 10.1093/nar/gkad275 PMC10320182

[43] Wu T , Chen J, Diao K, Wang G, Wang J, Yao H, . Neodb: a comprehensive neoantigen database and discovery platform for cancer immunotherapy. Database 2023;2023:baad041. 37311149 10.1093/database/baad041 PMC10263465

[44] Parra-Herran C , Lerner-Ellis J, Xu B, Khalouei S, Bassiouny D, Cesari M, . Molecular-based classification algorithm for endometrial carcinoma categorizes ovarian endometrioid carcinoma into prognostically significant groups. Mod Pathol 2017;30:1748–59. 28776572 10.1038/modpathol.2017.81

[45] van 't Erve I , Wesdorp NJ, Medina JE, Ferreira L, Leal A, Huiskens J, . KRAS A146 mutations are associated with distinct clinical behavior in patients with colorectal liver metastases. JCO Precis Oncol 2021;5:PO.21.00223. 34820593 10.1200/PO.21.00223 PMC8608264

[46] Kim B , Kang SY, Kim D, Heo YJ, Kim KM. PTEN protein loss and loss-of-function mutations in gastric cancers: the relationship with microsatellite instability, EBV, HER2, and PD-L1 expression. Cancers 2020;12:1724. 32610572 10.3390/cancers12071724 PMC7407887

[47] Post KL , Belmadani M, Ganguly P, Meili F, Dingwall R, McDiarmid TA, . Multi-model functionalization of disease-associated PTEN missense mutations identifies multiple molecular mechanisms underlying protein dysfunction. Nat Commun 2020;11:2073. 32350270 10.1038/s41467-020-15943-0 PMC7190743

[48] Xu J , Li Z, Wang J, Chen H, Fang JY. Combined PTEN mutation and protein expression associate with overall and disease-free survival of glioblastoma patients. Transl Oncol 2014;7:196–205. 24721394 10.1016/j.tranon.2014.02.004 PMC4101389

[49] Serebriiskii IG , Pavlov V, Tricarico R, Andrianov G, Nicolas E, Parker MI, . Comprehensive characterization of PTEN mutational profile in a series of 34,129 colorectal cancers. Nat Commun 2022;13:1618. 35338148 10.1038/s41467-022-29227-2 PMC8956741

[50] Rudd ML , Price JC, Fogoros S, Godwin AK, Sgroi DC, Merino MJ, . A unique spectrum of somatic PIK3CA (p110alpha) mutations within primary endometrial carcinomas. Clin Cancer Res 2011;17:1331–40. 21266528 10.1158/1078-0432.CCR-10-0540 PMC3060282

[51] Dahl JM , Thomas N, Tracy MA, Hearn BL, Perera L, Kennedy Scott R, . Probing the mechanisms of two exonuclease domain mutators of DNA polymerase ϵ. Nucleic Acids Res 2022;50:962–74. 35037018 10.1093/nar/gkab1255 PMC8789060

[52] Park VS , Pursell ZF. POLE proofreading defects: Contributions to mutagenesis and cancer. DNA Repair 2019;76:50–9. 30818169 10.1016/j.dnarep.2019.02.007 PMC6467506

[53] Príncipe C , Dionísio de Sousa IJ, Prazeres H, Soares P, Lima RT. LRP1B: a giant lost in cancer translation. Pharmaceuticals 2021;14:836. 34577535 10.3390/ph14090836 PMC8469001

[54] Lu M , Zhao B, Liu M, Wu L, Li Y, Zhai Y, . Pan-cancer analysis of SETD2 mutation and its association with the efficacy of immunotherapy. NPJ Precis Oncol 2021;5:51. 34127768 10.1038/s41698-021-00193-0 PMC8203790

[55] Okamura R , Kato S, Lee S, Jimenez RE, Sicklick JK, Kurzrock R. ARID1A alterations function as a biomarker for longer progression-free survival after anti-PD-1/PD-L1 immunotherapy. J Immunother Cancer 2020;8:e000438. 32111729 10.1136/jitc-2019-000438 PMC7057434

[56] Chen G , Chen P, Zhou J, Luo G. Pan-cancer analysis of histone methyltransferase KMT2D with potential implications for prognosis and immunotherapy in human cancer. Comb Chem High Throughput Screen 2023;26:83–92. 35189794 10.2174/1386207325666220221092318

[57] Scott RJ , Crooks R, Rose L, Attia J, Thakkinstian A, Thomas L, . Germline missense changes in the APC gene and their relationship to disease. Hered Cancer Clin Pract 2004;2:81–91. 20233475 10.1186/1897-4287-2-2-81 PMC2839999

[58] Zhang L , Shay JW. Multiple roles of APC and its therapeutic implications in colorectal cancer. J Natl Cancer Inst 2017;109:djw332. 28423402 10.1093/jnci/djw332 PMC5963831

[59] Lüchtenborg M , Weijenberg MP, Roemen GM, de Bruïne AP, van den Brandt PA, Lentjes MH, . APC mutations in sporadic colorectal carcinomas from The Netherlands Cohort Study. Carcinogenesis 2004;25:1219–26. 14976131 10.1093/carcin/bgh117

[60] De Mattos-Arruda L , Vazquez M, Finotello F, Lepore R, Porta E, Hundal J, . Neoantigen prediction and computational perspectives towards clinical benefit: recommendations from the ESMO Precision Medicine Working Group. Ann Oncol 2020;31:978–90. 32610166 10.1016/j.annonc.2020.05.008 PMC7885309

[61] Parkash V , Kulkarni Y, Ter Beek J, Shcherbakova PV, Kamerlin SCL, Johansson E. Structural consequence of the most frequently recurring cancer-associated substitution in DNA polymerase ε. Nat Commun 2019;10:373. 30670696 10.1038/s41467-018-08114-9 PMC6342957

[62] Cao H , Wang J, He L, Qi Y, Zhang JZ. DeepDDG: predicting the stability change of protein point mutations using neural networks. J Chem Inf Model 2019;59:1508–14. 30759982 10.1021/acs.jcim.8b00697

[63] Xing X , Kane DP, Bulock CR, Moore EA, Sharma S, Chabes A, . A recurrent cancer-associated substitution in DNA polymerase ε produces a hyperactive enzyme. Nat Commun 2019;10:374. 30670691 10.1038/s41467-018-08145-2 PMC6343027

