# Correction: Exploring Co-occurring POLE Exonuclease and Non-exonuclease Domain Mutations and Their Impact on Tumor Mutagenicity

**DOI:** 10.1158/2767-9764.CRC-24-0280

**Published:** 2024-05-31

**Authors:** Shreya M. Shah, Elena V. Demidova, Salena Ringenbach, Bulat Faezov, Mark Andrake, Arjun Gandhi, Pilar Mur, Julen Viana-Errasti, Joanne Xiu, Jeffrey Swensen, Laura Valle, Roland L. Dunbrack, Michael J. Hall, Sanjeevani Arora

In the original version of this article (1), the authors note a labeling error in two figures (Fig. 3 and Supplementary Fig. S3), which carried over to Supplementary Table and article text. The error does not affect the data or the conclusions presented in the article. This error has been corrected in the latest online HTML and PDF versions of the article. The authors regret this error.
