# Supplementary material for: Exploring Co-occurring POLE Exonuclease and Non-exonuclease Domain Mutations and Their Impact on Tumor Mutagenicity
Source: Cancer Res Commun. 2024 Jan 26;4(1):213–25. doi: 10.1158/2767-9764.CRC-23-0312 (PMC10812383; doi:10.1158/2767-9764.CRC-23-0312)
Supplement: Supplementary Data — All Supplementary_Legends_Figures_and_most Tables [file crc-23-0312-s01.docx]

**­Supplementary.**

**Supplementary References for Figure Legends.**

1 Hodel, K. P. *et al.* POLE Mutation Spectra Are Shaped by the Mutant Allele Identity, Its Abundance, and Mismatch Repair Status. *Mol Cell* **78**, 1166-1177.e1166, doi:10.1016/j.molcel.2020.05.012 (2020).

2 Kim, J. Y., Bang, H., Noh, S. J. & Choi, J. K. DeepNeo: a webserver for predicting immunogenic neoantigens. *Nucleic Acids Res* **51**, W134-w140, doi:10.1093/nar/gkad275 (2023).

3 Yuan, Z., Georgescu, R., Schauer, G. D., O'Donnell, M. E. & Li, H. Structure of the polymerase ε holoenzyme and atomic model of the leading strand replisome. *Nat Commun* **11**, 3156, doi:10.1038/s41467-020-16910-5 (2020).

4 Hogg, M. *et al.* Structural basis for processive DNA synthesis by yeast DNA polymerase ɛ. *Nat Struct Mol Biol* **21**, 49-55, doi:10.1038/nsmb.2712 (2014).

5 Jumper, J. *et al.* Highly accurate protein structure prediction with AlphaFold. *Nature* **596**, 583-589, doi:10.1038/s41586-021-03819-2 (2021).

6 Tunyasuvunakool, K. *et al.* Highly accurate protein structure prediction for the human proteome. *Nature* **596**, 590-596, doi:10.1038/s41586-021-03828-1 (2021).

7 Yuan, Z., Georgescu, R., Schauer, G. D., O’Donnell, M. E. & Li, H. Structure of the polymerase ε holoenzyme and atomic model of the leading strand replisome. *Nature Communications* **11**, 3156, doi:10.1038/s41467-020-16910-5 (2020).

8 Baranovskiy, A. G. *et al.* Crystal structure of the human Polϵ B-subunit in complex with the C-terminal domain of the catalytic subunit. *J Biol Chem* **292**, 15717-15730, doi:10.1074/jbc.M117.792705 (2017).

**Supplementary Figure Legends**

**Supplementary Figure 1.** COSMIC mutation signatures in Group 3 tumors. **A**, and **B.** The two major COSMIC mutational signatures associated with POLE ExoD driver defects (SBS 10a, and SBS 10b) were assessed ^1^. The signature sequence contexts for SBS 10a and 10b are shown as percentage of single base substitutions for cohorts in CRC and EC data.

**Supplementary Figure 2. A.** Overall three nucleotide sequence context of *POLE* variants in Group 3 tumors. All COSMIC mutational signatures associated with POLE ExoD driver defects (SBS 10a, SBS 10b, SBS 14, and SBS 28) were assessed ^1^. Pie chart distribution of SBS 10a, SBS 10b, SBS 14, and SBS 28 in CRC**,** EC**,** and OC are combined. Each of these signatures has a primary mutation which has been described as a “hotspot” ^1^; SBS 10a is C>A in TCT context; SBS 10b is C>T in the TCG context; SBS 14 is C>A in the NCT context (N is any base); and SBS 28 is T>G in the TTT context. In addition to these primary “hotspots”, all mutations that comprise >1% of the genome signature of interest were counted, capturing 88-90% of each signature in the analysis. **B.** *POLE* variants in Group 4 MSS TMB-H subset. *POLE* variants found in MSS CRC (round symbol) and EC (triangle symbol) tumor profiles. *POLE* variants are missense (in green), nonsense (in lavender), and any other (in fuchsia), TMB data, in bold in brackets for each *POLE* variant. The annotated POLE regions are NTD (grey, 31-281), ExoD (wheat, 282-527), polymerase (pink, palm: 528-950; cyan, fingers: 769-833; green, thumb: 951-1186), and CTL (dark grey, 1308-2222).

**Supplemental Figure 3.** Molecular features of OC. The mutational landscape and patient demographic/clinical characteristics (PDL1 by IHC, MSI Comprehensive, age, and sex) of the 4 cohorts for each cancer type were plotted using the GenVisR package of R.

**Supplementary Figure 4.** TMB versus Neoantigen prediction. The predicted neoantigen burden from the missense mutations in the top 10% of mutated genes that were exclusive to Group 3. The computed neoantigen burden from DeepNeo ^2^ was compared with tumor mutation burden per tumor, graphed in GraphPad Prism. Spearman rank with P<0.05 considered statistically significant for each comparison.

**Supplementary Figure 5.** **A**, and **B.** Comparison of mTMB and ΔΔG values in Group 2 and 3 tumors in the Caris dataset. With AlphaFold2 DNA bound model and Rosetta ddG_monomer, we generated 25 repacked decoys for each mutation and compared the average energy score for these decoys to an average for 25 decoys of the wildtype protein. We used a cutoff of ±1.45 kcal/mol for significant ΔΔG, corresponding to ~2 standard deviations of the differences of the mean Rosetta scores for WT and mutant structures. **A.** Comparison of mTMB and ΔΔG values in Group 2 and 3 tumors by the number of *POLE* Variants. Data for CRC, EC, and OC genomic profiles were combined and ΔΔG values are plotted against the mTMB. For Group 2 or the Group 3 data with + 1 *POLE* variant plots, each filled round circle represents a single tumor genomic profile. **B.** Comparison of mTMB and ΔΔG values in Group 2 and 3 tumors by *POLE* ExoD driver. Data for CRC, EC, and OC genomic profiles were combined and ΔΔG values are plotted against the mTMB. **A and B**. Group 3 tumors with multiple variants, a circle next to another circle (without any space) represents a single tumor. For clarity, ΔΔG values for ExoD drivers in Group 3 tumors are not shown (they are same as in Group 2). Color in each filled circle- green or shades of green, structure-destabilizing variants (positive ΔΔG); white, variants that are within the standard deviations of ±1.45 kcal/mol and are structure neutral; red or shades of red, structure-stabilizing variants (negative ΔΔG). Yellow, nonsense, or frameshift variants; Black, ΔΔG values not calculated in the with DNA model.

**Supplementary Figure 6.** Structure-function assessment of *POLE* variants in Group 2, 3, and 4 tumors (CLS dataset). Human POLE structure models based on yeast POLE templates without DNA bound (full-length yeast POLE with Dbp2, Dbp3, and Dbp4 subunits, PDB:6WJV ^3^ and with DNA bound (N-terminal lobe only, PDB:4M8O ^4^ and were generated using AlphaFold2 ^5,6^. **A**. The length of the POLE protein is 2286 amino acids (aa). The structure can be divided into the NTL (aa 1-1186, blue) and the CTL (aa 1308-2286, orange), which are connected via a linker (aa 1187-1307, mostly weakly predicted, shown in gray, except for an interdomain helix in magenta, aa 1264-1292). **B.** The NTL contains the NTD (aa 31-269, yellow), the ExoD (aa 268-527, blue), and the polymerase domain (aa 528-1186, green). The polymerase domain is further divided into the palm (aa 528-970), the structurally “flexible” part of the palm called the finger (aa 753-833), and the thumb (aa 951-1186) ^7,8^. The ExoD and polymerase domains together (aa 270-1186) perform DNA synthesis and repair. The roles of the NTD of the NTL and the CTL are not well-studied; the CTL stabilizes the POLE structure and interacts with other subunits in the POLE holoenzyme complex (Dpb2, Dpb3, and Dpb4) ^7^. **C**. Structural context of the ExoD driver mutations (n=20). None of the residues are in direct contact with DNA, as determined by superposing the AlphaFold2 model based on PDB:4M8O with the yeast POLE/DNA complex structure in PDB:4M8O. **D**. Structural context of the POLE CTL variants. **E.** Structural context of the POLE N-terminal variants (co-occurring with V411L driver). The 7 variants in the V411L + one variants Group 3 tumors associated with higher mTMB are scattered across the full length of POLE (K122N in the NTD, and P370T in the ExoD domain, A788V and D860G in the polymerase domain, R1233* and Q1239R in the linker, and R2131C in the CTD). P370T and D860G in the NTL are destabilizing according to the ddG_monomer. **F, G**. Assessment of variants from the NTD subdomain and the ExoD with striking (highly destabilizing or stabilizing) ΔΔG values.

**Supplementary Table Legends.**

**Supplementary Table 1.** All patient clinical, demographic, and molecular data in this manuscript (de-identified). The data are segregated by Groups 1-4 for each cancer type (CRC, EC, OC); all mutational data represented in Figures 4A-B and Supplemental Figure 5 is provided; mutational counts for specific mutations in KRAS, PTEN and PIK3CA for Figures 4A-B with corresponding POLE mutation signature context for KRAS A146T, PTEN R130Q, and PIK3CA R88Q (excel file; multiple sheets).

**Supplementary Table 2.** Age Distribution of CRC, EC, and OC patients with *POLE*-mutated tumors.

**Supplementary Table 3.** mTMB comparisons in the Caris Life Sciences dataset.

**Supplementary Table 4.** mTMB comparisons in TCGA dataset.

**Supplementary Table 5.** Neoantigens predictions for Group 3 tumor mutations by DeepNeo (sheet 1 for CRC; sheet 2 for EC) and Neodb (sheet 3 for CRC, and EC). Sheet 1 and 2 for DeepNeo data also show the TMB per MB data against the predicted neoantigen burden. Subsequent sub sheets show raw neoantigen prediction data by DeepNeo.

**Supplementary Table 6.** Rosetta ΔΔG values for POLE variants and drivers with or without DNA (excel file).

**Supplementary Table 7.** Mutations in Group 3 tumors with P286R or V411L plus one variant and mTMB comparisons.


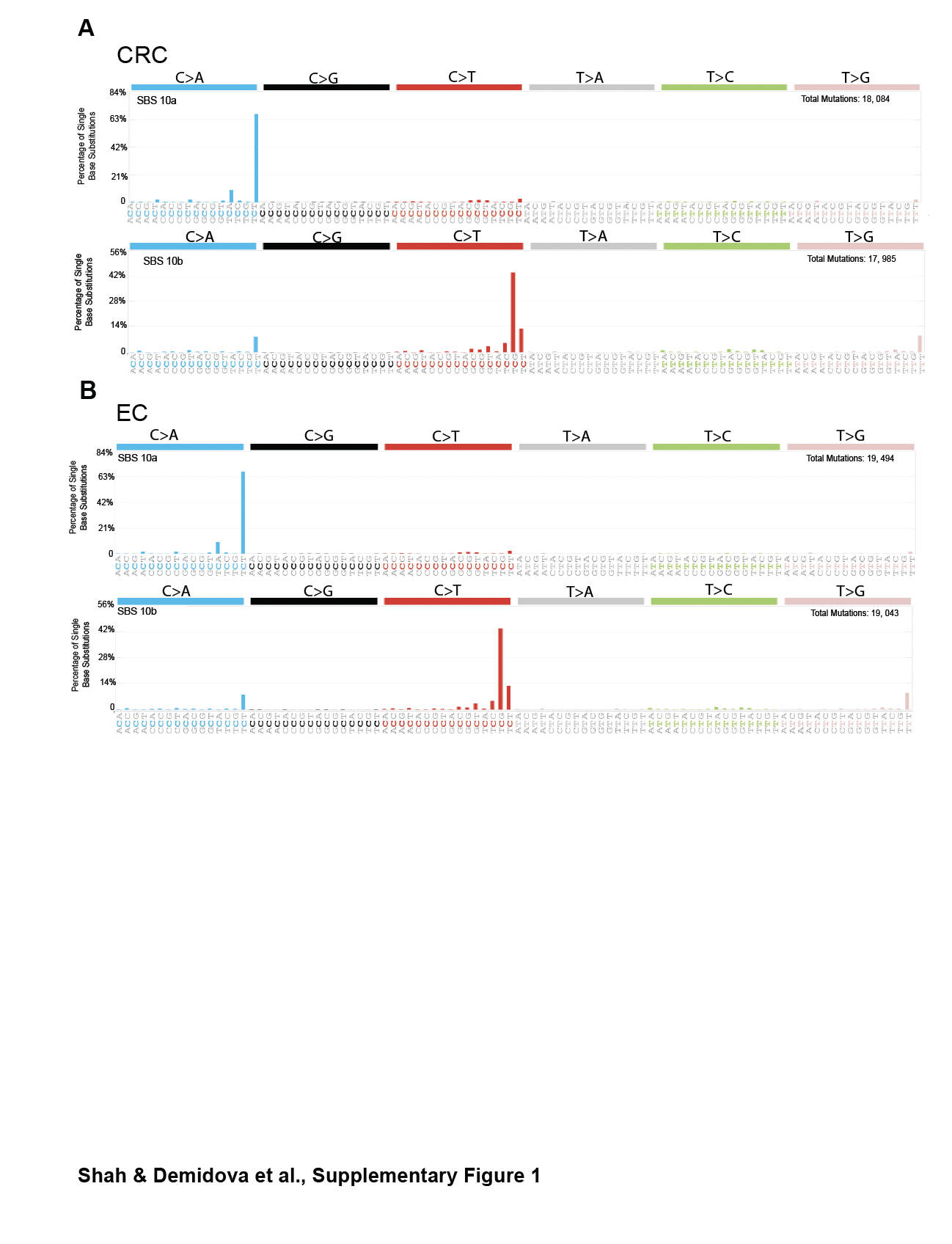


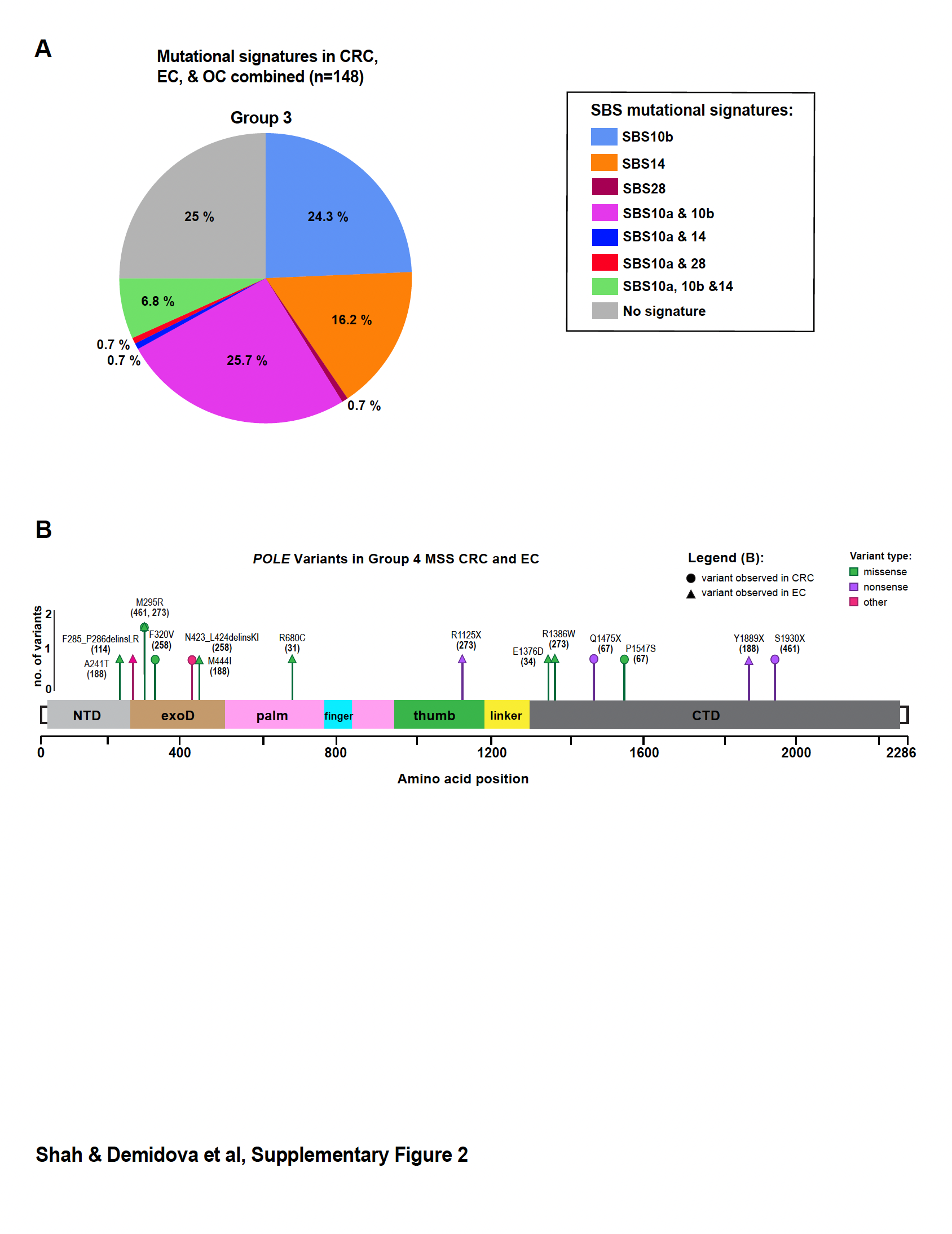


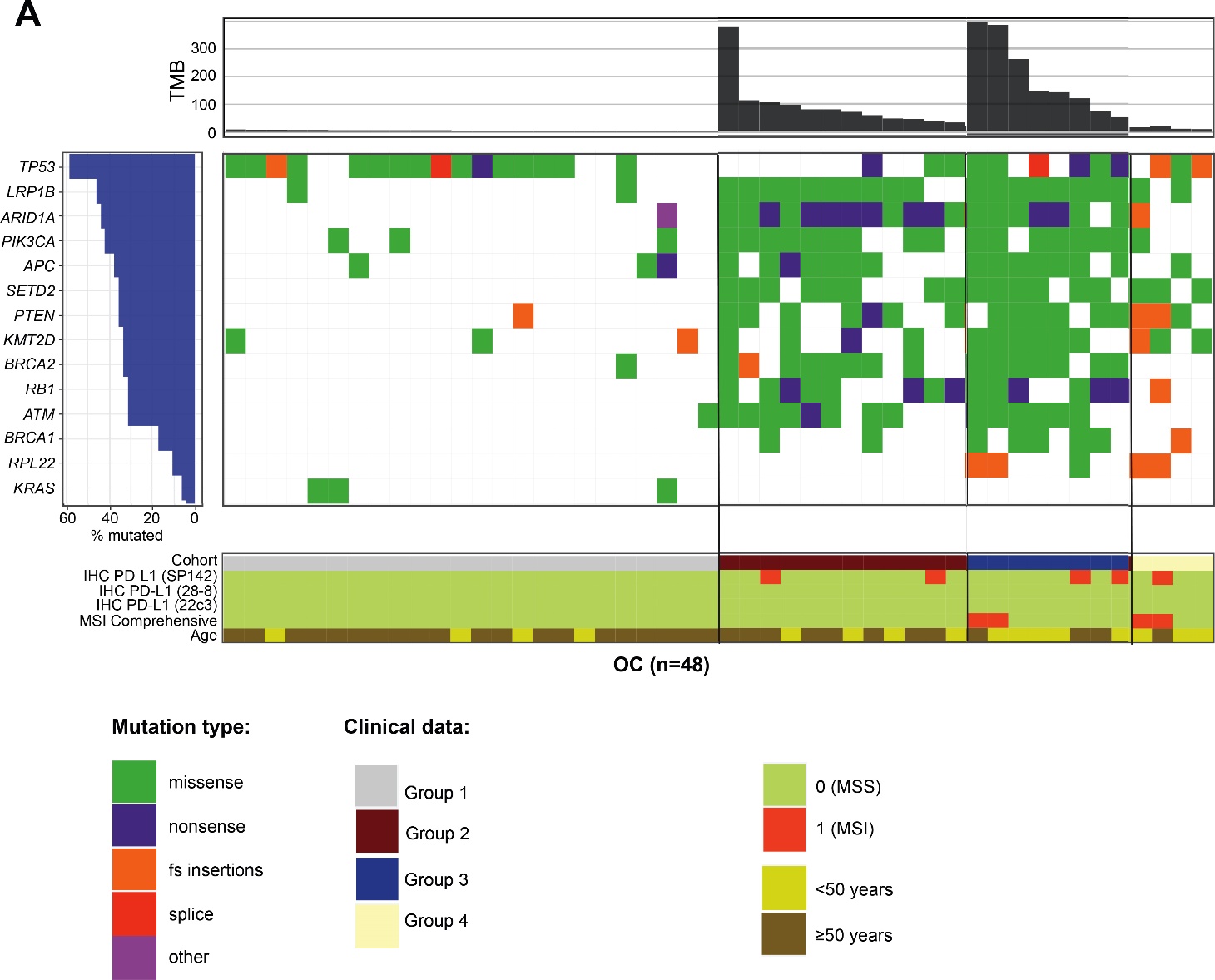


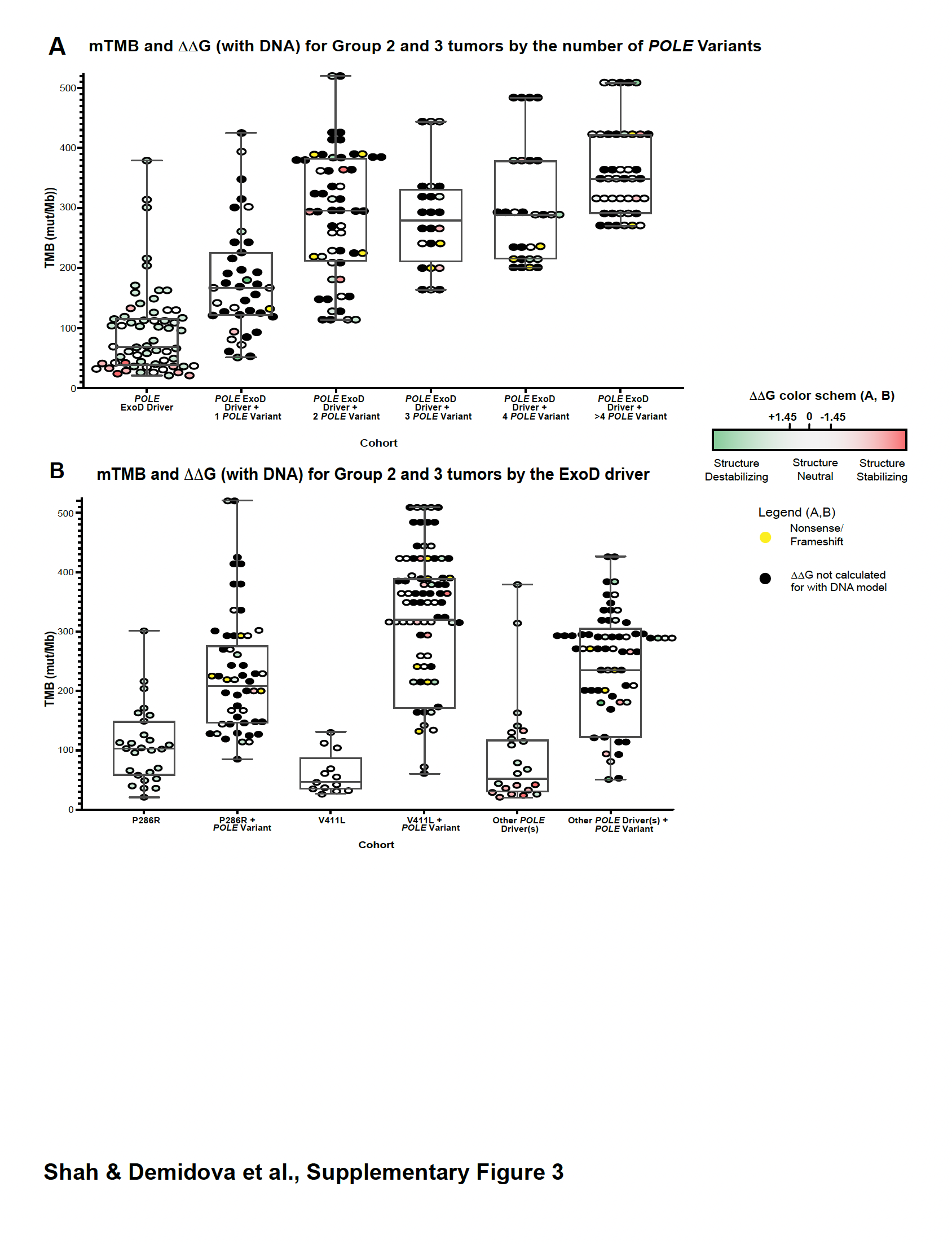


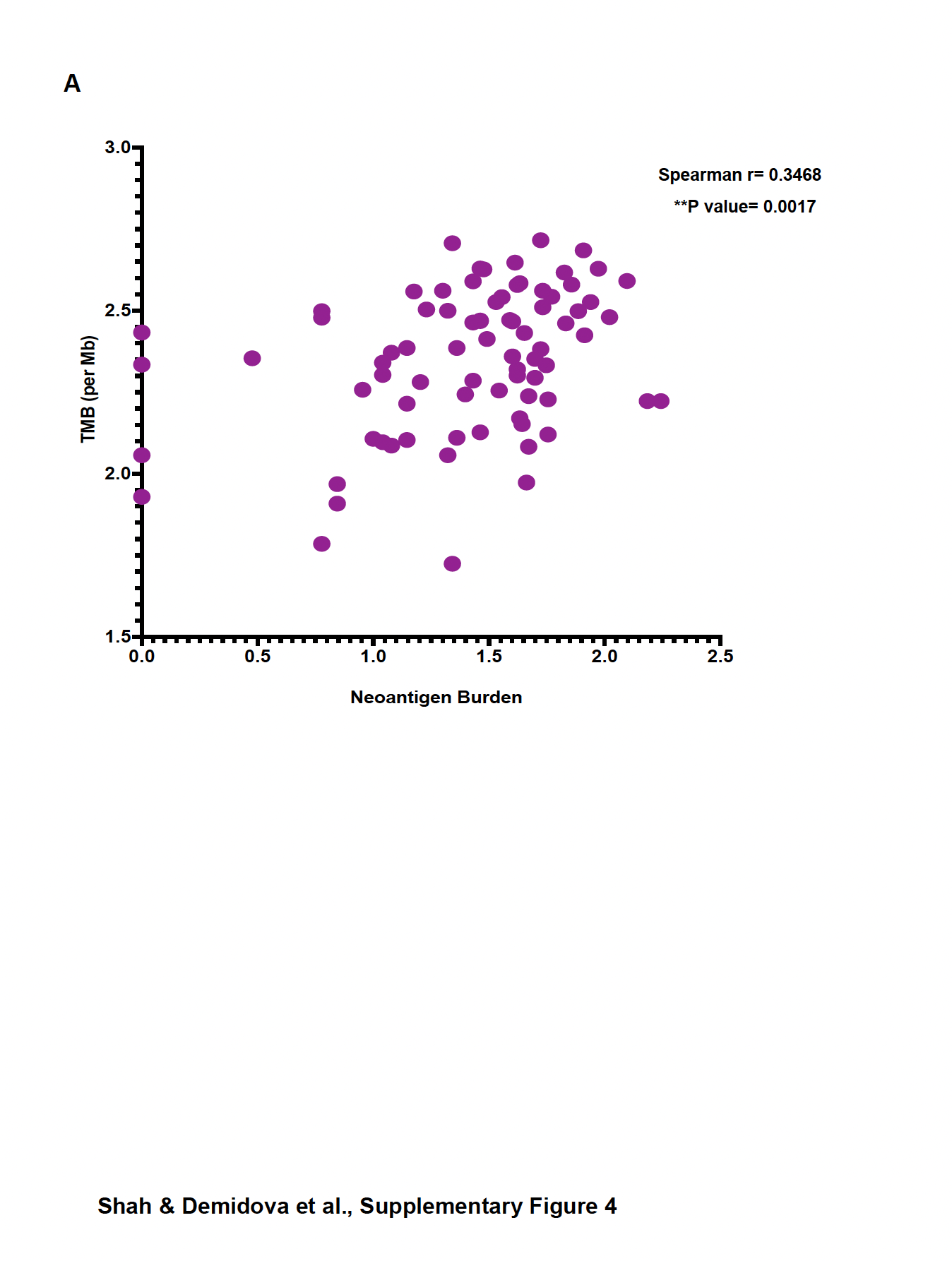


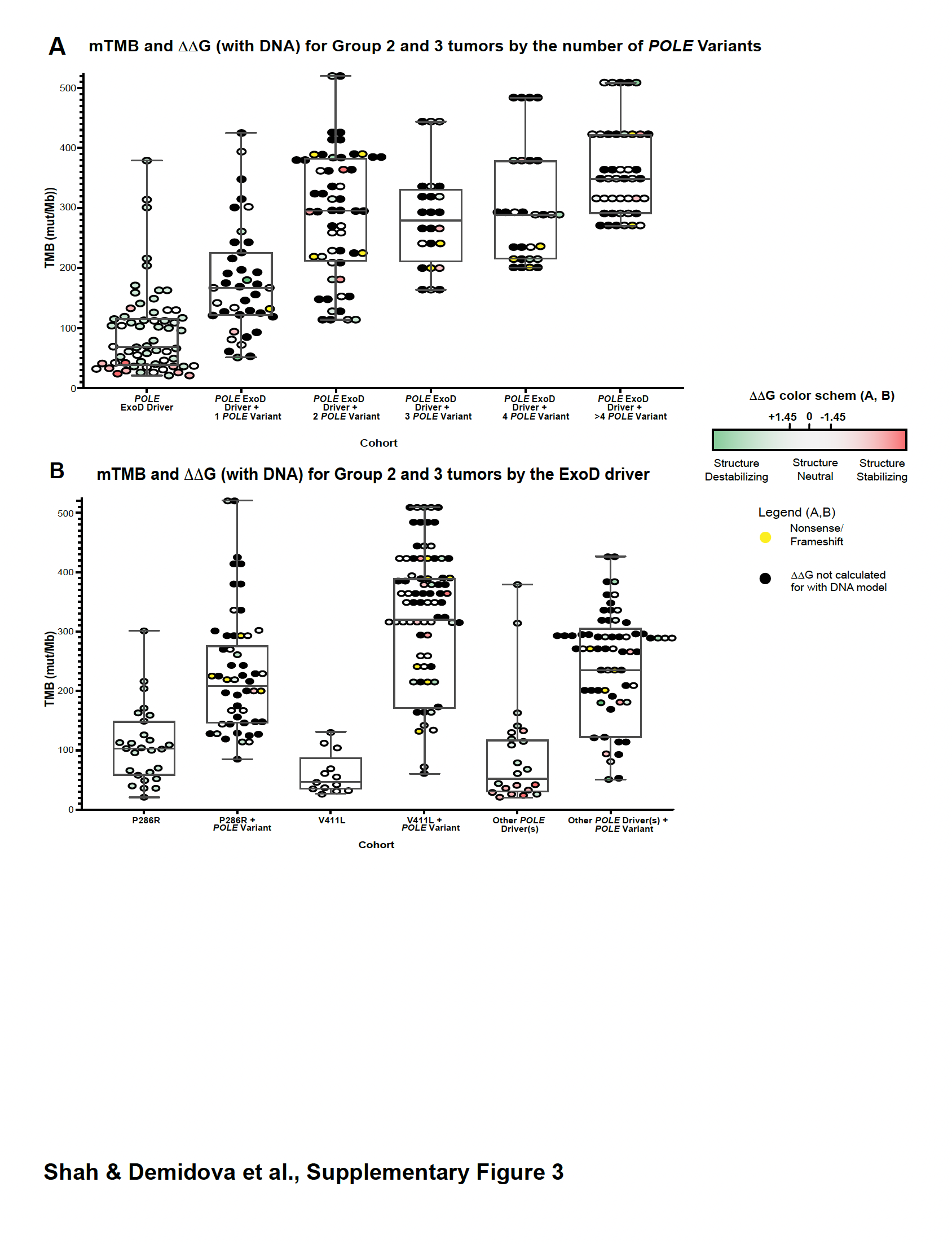


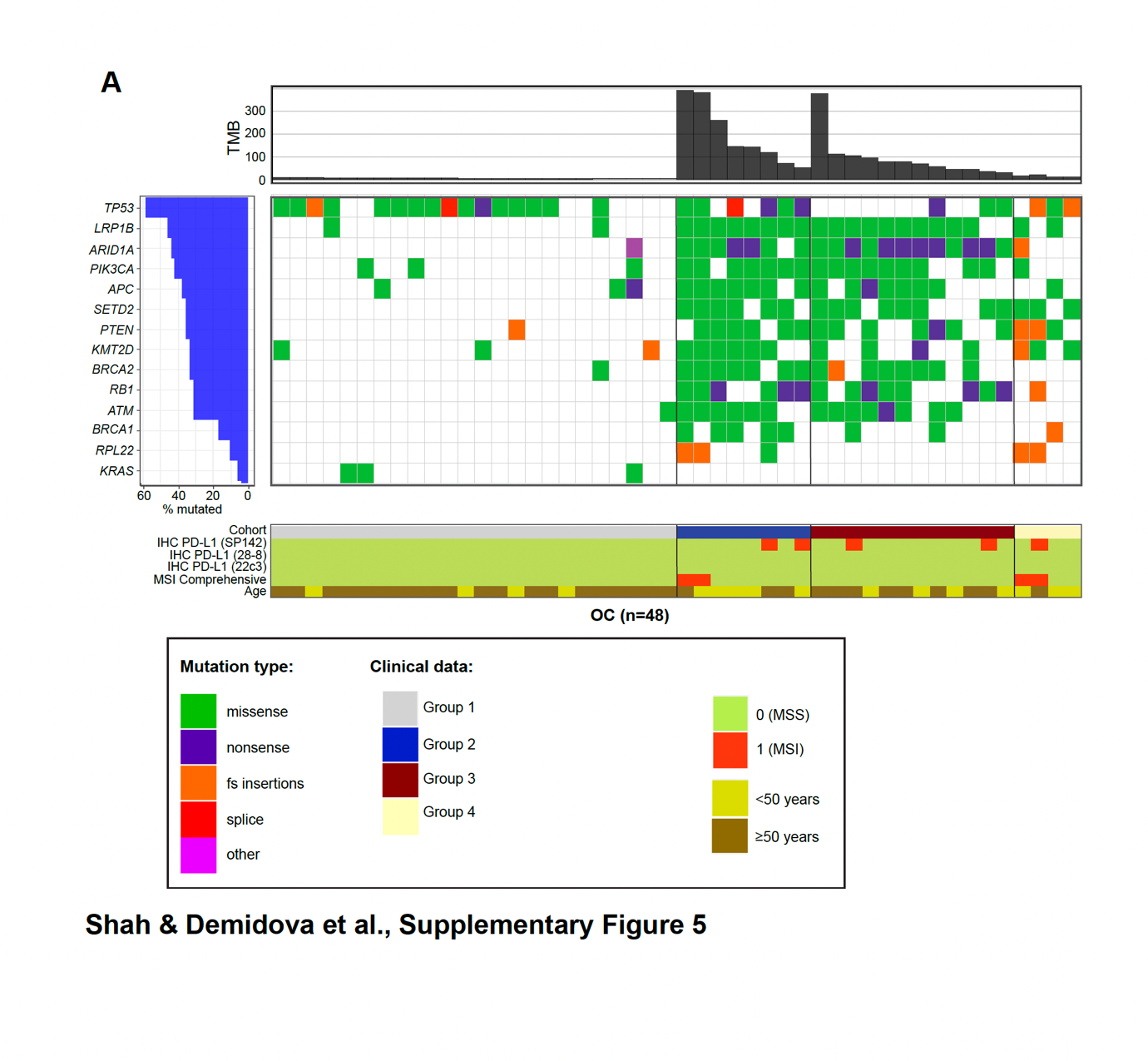


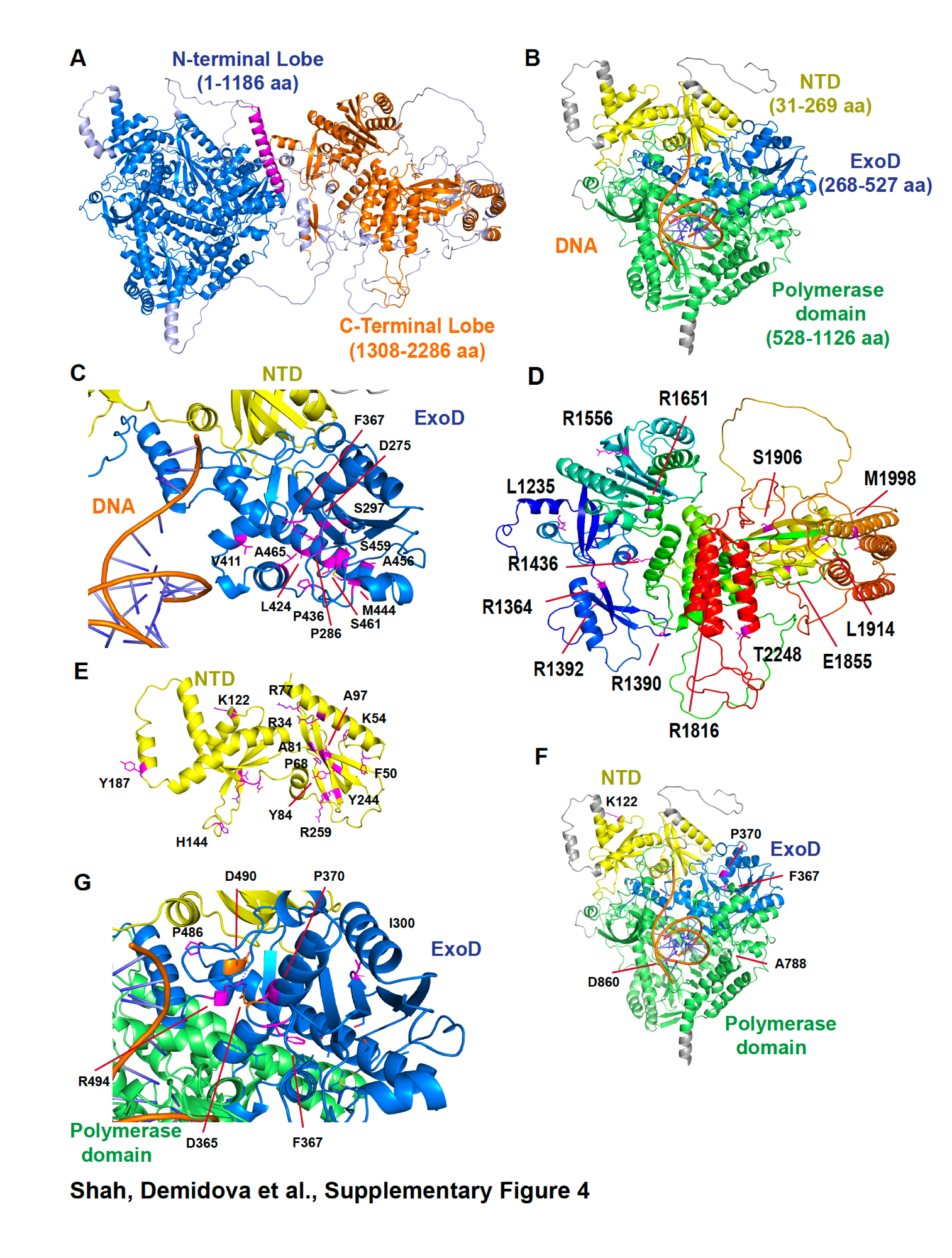


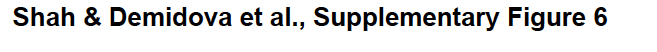


**Supplementary Table 2.** Age Distribution of CRC, EC, and OC patients with *POLE*-mutated tumors.

|  | **Age Distribution of Patients** | | | | | |
| --- | --- | --- | --- | --- | --- | --- |
|  | Q0 | Q1 | Median | Q3 | Q4 | Mean |
|  | **Colorectal Cancer** | | | | | |
| Group 1 | 28 | 52 | 56 | 63.75 | 84 | 57.72 |
| Group 2 | 24 | 40 | 53 | 62 | 69 | 49.64 |
| Group 3 | 28 | 40 | 45.5 | 57.75 | 73 | 48.08 |
| Group 4 | 25 | 37.5 | 56 | 66.5 | 76 | 52.67 |
|  | **Endometrial Cancer** | | | | | |
| Group 1 | 22 | 58 | 62 | 70 | 87 | 63.46 |
| Group 2 | 37 | 52 | 57 | 66 | 88 | 58.81 |
| Group 3 | 34 | 52.5 | 58 | 64 | 84 | 58.44 |
| Group 4 | 31 | 59.75 | 66 | 70.25 | 88 | 65.47 |
|  | **Ovarian Cancer** | | | | | |
| Group 1 | 44 | 57.25 | 62.50 | 72.25 | 83 | 63.08 |
| Group 2 | 32 | 43 | 51.5 | 55.25 | 59 | 49.42 |
| Group 3 | 34 | 43.5 | 48.5 | 57.75 | 63 | 49.38 |
| Group 4 | 38 | 40.5 | 48 | 51 | 52 | 46.50 |
|  | **All Cancers** | | | | | |
| Group 1 | 22 | 57 | 62 | 70 | 87 | 62.07 |
| Group 2 | 24 | 49.25 | 55.5 | 62 | 88 | 55.25 |
| Group 3 | 28 | 47.5 | 55 | 62 | 84 | 54.83 |
| Group 4 | 25 | 58 | 65 | 70 | 88 | 63.06 |

**Supplementary Table 3.** mTMB comparisons in the Caris Life Sciences dataset.

| **Caris data set** | **Group 1: *POLE* variant**  **TMB-L** | | | **Group 2: *POLE* ExoD driver** | | | **Group 3: *POLE* ExoD driver + *POLE* Variant** | | |
| --- | --- | --- | --- | --- | --- | --- | --- | --- | --- |
| Cancer type | **CRC** | **EC** | **OC** | **CRC** | **EC** | **OC** | **CRC** | **EC** | **OC** |
| mTMB (range),  including MSI & MSS | 6.0  (3-9) | 7  (3-9) | 5  (4-9) | 115  (61-216) | 52  (21-314) | 69  (31-379) | 264.5  (114-414) | 219  (53-520) | 145  (51-394) |
| Statistics | ******* | ******* | ******* |  |  |  | ***** | ***** | * |
| mTMB (range),  excluding MSI | 6.0  (3-9) | 7  (3-9) | 5  (4-9) | 115  (61-216) | 52  (21-301) | 69  (31-379) | 259  (114-414) | 181  (53-520) | 131.5  (51-261) |
| Statistics | ******* | ******* | ******* |  |  |  | ***** | ***** | NS |

CRC, colorectal cancer; EC, endometrial cancer; OC, ovarian cancer.

*** represents p<0.001 obtained from Mann-Whitney test. * is p<0.05, *** is p<0.001. NS is not significant. Group 2 was compared with Group 1 and Group 3.

**Supplementary Table 4.** mTMB comparisons in TCGA dataset.

| **TCGA data set** | **Group 2: *POLE* ExoD driver** | **Group 3: *POLE* ExoD driver + *POLE* Variant** |
| --- | --- | --- |
| mTMB (range), n  including MSI & MSS | 104.6 (28.2-302.9), 17 | 250.4 (58.3-532.7), 29 |
| Statistics |  | *** |
| mTMB (range),  excluding MSI | 108.7(28.2-302.9), 16 | 272.1 (58.3-532.7), 24 |
| Statistics |  | *** |

*** is p<0.001 obtained from Mann Whitney test.

**Supplementary Table 7.** Mutations in Group 3 tumors with P286R or V411L plus one variant and mTMB comparisons.

| **Mutations in Group 3 tumors with P286R + one Variant** | **mTMB** |
| --- | --- |
| E1855D | 129 |
| F990C | 261 |
| L1235I | 197 |
| L1914I | 156 |
| M1998I | 85 |
| R1364C | 425 |
| R1382C | 301 |
| R1390C | 243 |
| R1436W | 146 |
| R1556W | 119 |
| R1651K | 125 |
| R1826W | 243 |
| R1826W | 175 |
| R2017C | 127 |
| R494W | 167 |
| R494W | 167 |
| R77C | 302 |
| S1906Y | 226 |
| S1906Y | 193 |
| T2248I | 216 |

| **Mutations in Group 3 tumors with V411L + one Variant** | **mTMB** |
| --- | --- |
| A788V | 134 |
| D860G | 394 |
| K122N | 142 |
| P370T | 72 |
| Q1239R | 61 |
| R1233* | 132 |
| R2131C | 173 |

mTMB, median Tumor Mutation Burden value.
