# Supplementary material for: Exploring Co-occurring POLE Exonuclease and Non-exonuclease Domain Mutations and Their Impact on Tumor Mutagenicity
Source: Cancer Res Commun. 2024 Jan 26;4(1):213–25. doi: 10.1158/2767-9764.CRC-23-0312 (PMC10812383; doi:10.1158/2767-9764.CRC-23-0312)
Supplement: Supplementary Table 2 — Age Distribution of CRC, EC, and OC patients with POLE-mutated tumors. [file crc-23-0312-s03.docx]

**Supplementary Table 2.** Age Distribution of CRC, EC, and OC patients with *POLE*-mutated tumors.

|  | **Age Distribution of Patients** | | | | | |
| --- | --- | --- | --- | --- | --- | --- |
|  | Q0 | Q1 | Median | Q3 | Q4 | Mean |
|  | **Colorectal Cancer** | | | | | |
| Group 1 | 28 | 52 | 56 | 63.75 | 84 | 57.72 |
| Group 2 | 24 | 40 | 53 | 62 | 69 | 49.64 |
| Group 3 | 28 | 40 | 45.5 | 57.75 | 73 | 48.08 |
| Group 4 | 25 | 37.5 | 56 | 66.5 | 76 | 52.67 |
|  | **Endometrial Cancer** | | | | | |
| Group 1 | 22 | 58 | 62 | 70 | 87 | 63.46 |
| Group 2 | 37 | 52 | 57 | 66 | 88 | 58.81 |
| Group 3 | 34 | 52.5 | 58 | 64 | 84 | 58.44 |
| Group 4 | 31 | 59.75 | 66 | 70.25 | 88 | 65.47 |
|  | **Ovarian Cancer** | | | | | |
| Group 1 | 44 | 57.25 | 62.50 | 72.25 | 83 | 63.08 |
| Group 2 | 32 | 43 | 51.5 | 55.25 | 59 | 49.42 |
| Group 3 | 34 | 43.5 | 48.5 | 57.75 | 63 | 49.38 |
| Group 4 | 38 | 40.5 | 48 | 51 | 52 | 46.50 |
|  | **All Cancers** | | | | | |
| Group 1 | 22 | 57 | 62 | 70 | 87 | 62.07 |
| Group 2 | 24 | 49.25 | 55.5 | 62 | 88 | 55.25 |
| Group 3 | 28 | 47.5 | 55 | 62 | 84 | 54.83 |
| Group 4 | 25 | 58 | 65 | 70 | 88 | 63.06 |
