# Supplementary material for: Exploring Co-occurring POLE Exonuclease and Non-exonuclease Domain Mutations and Their Impact on Tumor Mutagenicity
Source: Cancer Res Commun. 2024 Jan 26;4(1):213–25. doi: 10.1158/2767-9764.CRC-23-0312 (PMC10812383; doi:10.1158/2767-9764.CRC-23-0312)
Supplement: Supplementary Table 3 — mTMB comparisons in the Caris Life Sciences dataset. [file crc-23-0312-s04.docx]

**Supplementary Table 3.** mTMB comparisons in the Caris Life Sciences dataset.

| **Caris data set** | **Group 1: *POLE* variant**  **TMB-L** | | | **Group 2: *POLE* ExoD driver** | | | **Group 3: *POLE* ExoD driver + *POLE* Variant** | | |
| --- | --- | --- | --- | --- | --- | --- | --- | --- | --- |
| Cancer type | **CRC** | **EC** | **OC** | **CRC** | **EC** | **OC** | **CRC** | **EC** | **OC** |
| mTMB (range),  including MSI & MSS | 6.0  (3-9) | 7  (3-9) | 5  (4-9) | 115  (61-216) | 52  (21-314) | 69  (31-379) | 264.5  (114-414) | 219  (53-520) | 145  (51-394) |
| Statistics | ******* | ******* | ******* |  |  |  | ***** | ***** | * |
| mTMB (range),  excluding MSI | 6.0  (3-9) | 7  (3-9) | 5  (4-9) | 115  (61-216) | 52  (21-301) | 69  (31-379) | 259  (114-414) | 181  (53-520) | 131.5  (51-261) |
| Statistics | ******* | ******* | ******* |  |  |  | ***** | ***** | NS |

CRC, colorectal cancer; EC, endometrial cancer; OC, ovarian cancer.

*** represents p<0.001 obtained from Mann-Whitney test. * is p<0.05, *** is p<0.001. NS is not significant. Group 2 was compared with Group 1 and Group 3.
