# Supplementary material for: Exploring Co-occurring POLE Exonuclease and Non-exonuclease Domain Mutations and Their Impact on Tumor Mutagenicity
Source: Cancer Res Commun. 2024 Jan 26;4(1):213–25. doi: 10.1158/2767-9764.CRC-23-0312 (PMC10812383; doi:10.1158/2767-9764.CRC-23-0312)
Supplement: Supplementary Table 4 — mTMB comparisons in TCGA dataset. [file crc-23-0312-s05.docx]

**Supplementary Table 4.** mTMB comparisons in TCGA dataset.

| **TCGA data set** | **Group 2: *POLE* ExoD driver** | **Group 3: *POLE* ExoD driver + *POLE* Variant** |
| --- | --- | --- |
| mTMB (range), n  including MSI & MSS | 104.6 (28.2-302.9), 17 | 250.4 (58.3-532.7), 29 |
| Statistics |  | *** |
| mTMB (range),  excluding MSI | 108.7(28.2-302.9), 16 | 272.1 (58.3-532.7), 24 |
| Statistics |  | *** |

*** is p<0.001 obtained from Mann Whitney test.
