# Supplementary material for: Exploring Co-occurring POLE Exonuclease and Non-exonuclease Domain Mutations and Their Impact on Tumor Mutagenicity
Source: Cancer Res Commun. 2024 Jan 26;4(1):213–25. doi: 10.1158/2767-9764.CRC-23-0312 (PMC10812383; doi:10.1158/2767-9764.CRC-23-0312)
Supplement: Supplementary Table 7 — Mutations in Group 3 tumors with P286R or V411L plus one variant and mTMB comparisons. [file crc-23-0312-s08.docx]

**Supplementary Table 7.** Mutations in Group 3 tumors with P286R or V411L plus one variant and mTMB comparisons.

| **Mutations in Group 3 tumors with P286R + one Variant** | **mTMB** |
| --- | --- |
| E1855D | 129 |
| F990C | 261 |
| L1235I | 197 |
| L1914I | 156 |
| M1998I | 85 |
| R1364C | 425 |
| R1382C | 301 |
| R1390C | 243 |
| R1436W | 146 |
| R1556W | 119 |
| R1651K | 125 |
| R1826W | 243 |
| R1826W | 175 |
| R2017C | 127 |
| R494W | 167 |
| R494W | 167 |
| R77C | 302 |
| S1906Y | 226 |
| S1906Y | 193 |
| T2248I | 216 |

| **Mutations in Group 3 tumors with V411L + one Variant** | **mTMB** |
| --- | --- |
| A788V | 134 |
| D860G | 394 |
| K122N | 142 |
| P370T | 72 |
| Q1239R | 61 |
| R1233* | 132 |
| R2131C | 173 |

mTMB, median Tumor Mutation Burden value.
